# Progress Toward a Large-Scale Synthesis of Molnupiravir (MK-4482, EIDD-2801) from Cytidine

**DOI:** 10.1021/acsomega.1c00772

**Published:** 2021-04-08

**Authors:** Grace P. Ahlqvist, Catherine P. McGeough, Chris Senanayake, Joseph D. Armstrong, Ajay Yadaw, Sarabindu Roy, Saeed Ahmad, David R. Snead, Timothy F. Jamison

**Affiliations:** † Department of Chemistry, Massachusetts Institute of Technology, 77 Massachusetts Avenue, Cambridge, Massachusetts 02139, United States; ‡ TCG GreenChem, Inc., Process R&D Center at Princeton South, 701 Charles Ewing Boulevard, Ewing, New Jersey 08628, United States; § Medicines for All Institute, 737 N. 5th Street, Box 980100, Richmond, Virginia 23298, United States

## Abstract

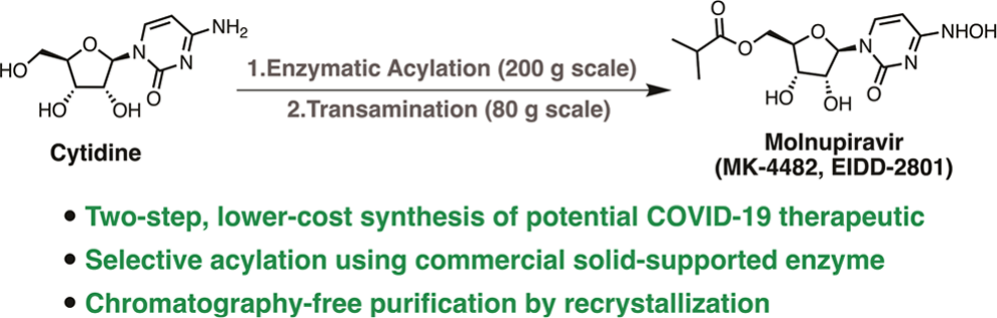

Molnupiravir (MK-4482, EIDD-2801) is a promising orally bioavailable drug candidate for the treatment of COVID-19. Herein, we describe a supply-centered and chromatography-free synthesis of molnupiravir from cytidine, consisting of two steps: a selective enzymatic acylation followed by transamination to yield the final drug product. Both steps have been successfully performed on a decagram scale: the first step at 200 g and the second step at 80 g. Overall, molnupiravir has been obtained in a 41% overall isolated yield compared to a maximum 17% isolated yield in the patented route. This route provides many advantages to the initial route described in the patent literature and would decrease the cost of this pharmaceutical should it prove safe and efficacious in ongoing clinical trials.

## Introduction

**Scheme 1:**
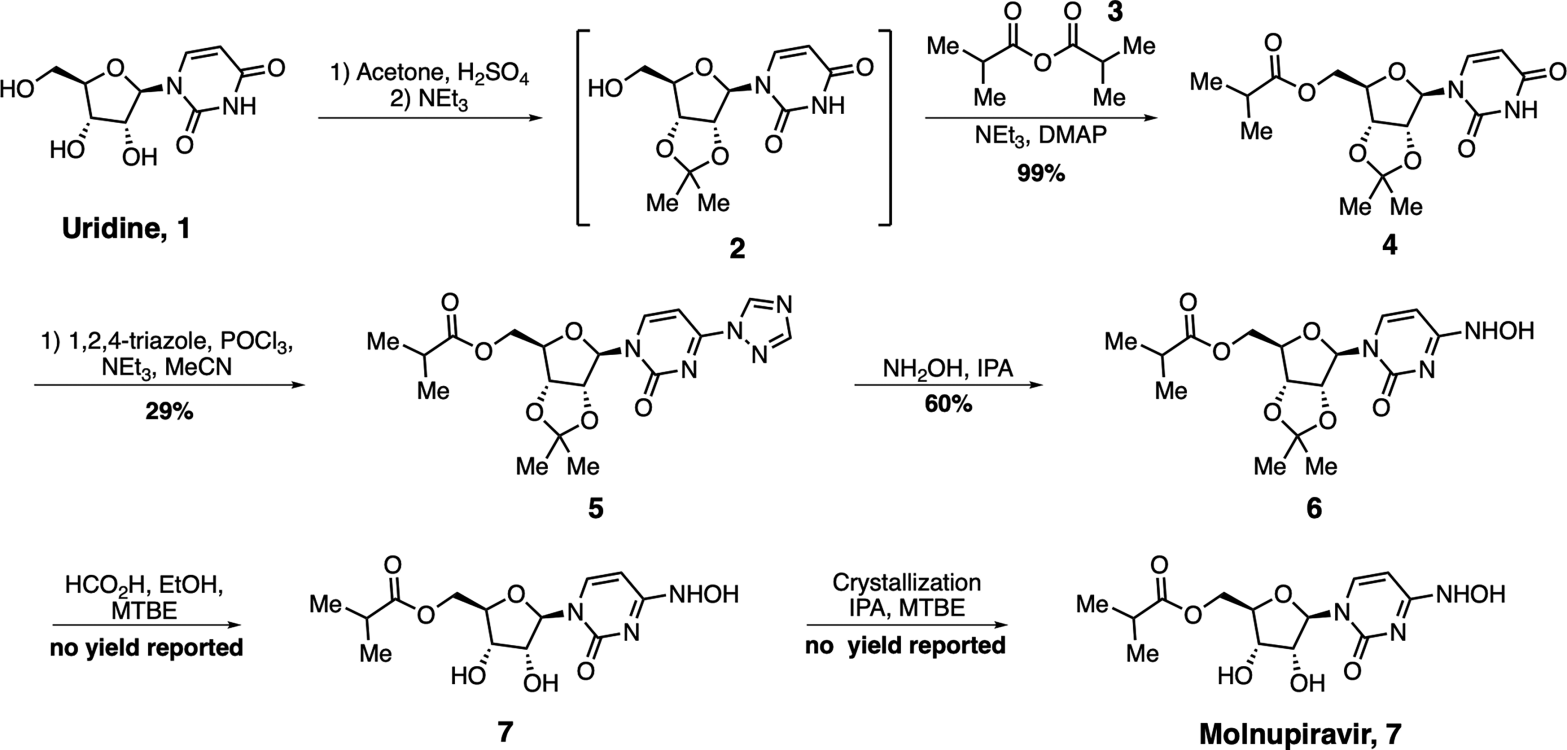
Synthetic Route of Molnupiravir Disclosed by Emory University in 2019

Molnupiravir (MK-4482, EIDD-2801) is in development by Merck after licensing from Ridgeback Biopharmaceuticals as an orally dosed antiviral for the treatment of COVID-19.^1^ Animal studies have shown successful inhibition of SARS-CoV-2^2^ as well as prevention of viral transmission.^3^ If shown to be safe and effective in ongoing clinical trials, this compound would be an important tool in the toolbox of physicians working to counter the effects of the SARS-CoV-2 virus pandemic. Drug availability, however, depends on the efficient and cost-effective synthesis of the drug molecule to ensure broad global access to this potentially valuable medication. The original synthetic route was disclosed by Emory University in 2019; this route made molnupiravir in five steps with uridine (**1**) as the starting material.^4^ Since the yield for the final two steps is not reported, this route has a 17% maximum overall yield (Scheme 1).

**Figure 1:**
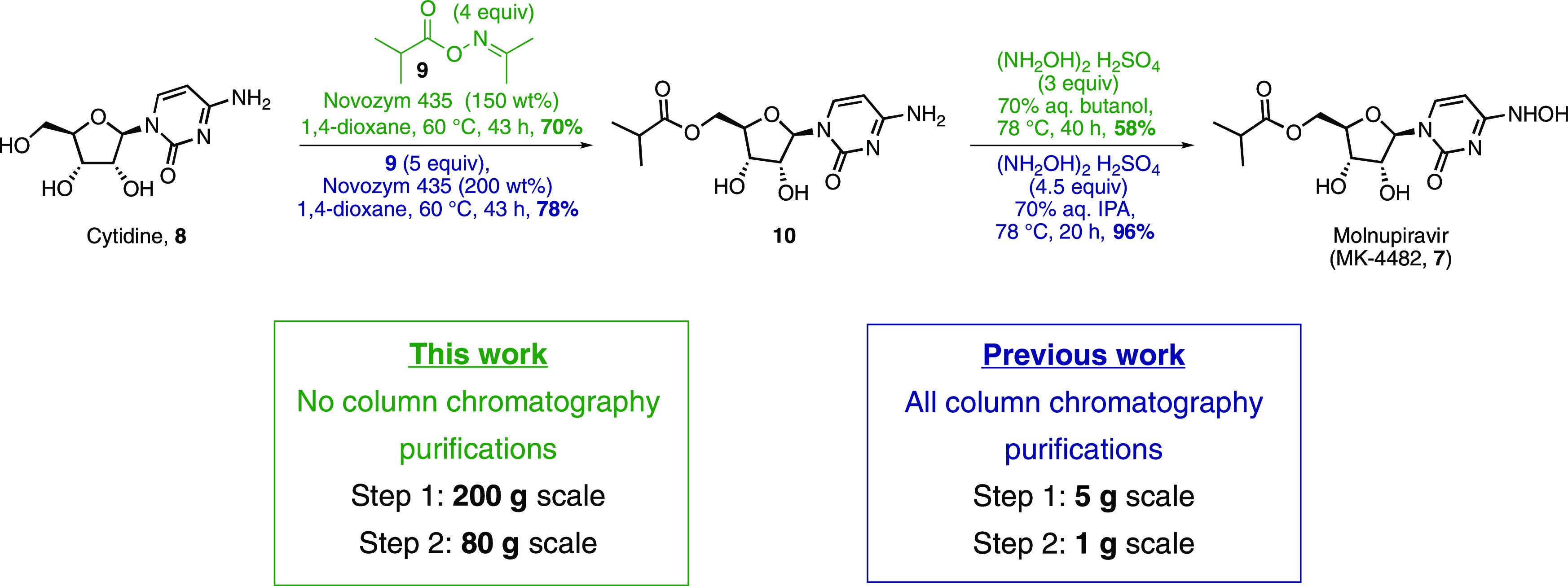
This work (green) compared to the previous work (blue).

We previously demonstrated the potential of a two-step route from cytidine (**8**) to molnupiravir (**7**), which has many advantages over the previously patented route including cost and the overall yield (Figure 1).^5^

However, several challenges prevent the previously reported route from being implemented at the manufacturing scale. Most notably, both the intermediate and the final active pharmaceutical ingredient (API) were purified by column chromatography, so we sought to develop alternate workup and crystallization procedures to provide a pure material without chromatographic purification. Other opportunities for improvement included examining the surprisingly high cost of acetone oxime and refining process parameters such as reaction concentration, catalyst loading, and stoichiometry. Thus, we set out to re-examine this route to enable this process to be run at an increased scale.

## Results and Discussion

The oxime ester acylating agent (**9**) proved uniquely suited to selective enzyme acylation (Supporting Information (SI) Section 2.1). Unfortunately, the acetone oxime used in the synthesis of the acylating agent proved to be a major cost driver of our initial route investigation (SI, Section 1).

**Scheme 2:**
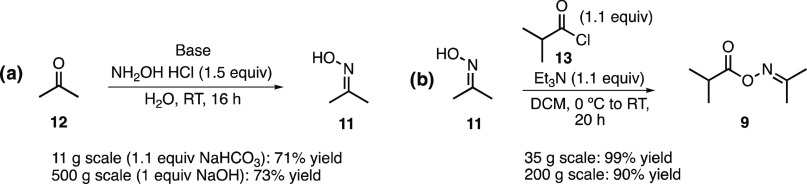
(a) Synthesis of Acetone Oxime (**11**); (b) Synthesis of Acylating Agent **9** Using Acetone Oxime (**11**) and Isobutyryl Chloride (**13**)

Surprised by the high cost of such a simple material, we sought to determine the cost of synthesizing this material ourselves. Gratifyingly, we found that a reported procedure^6^ for acetone oxime synthesis provided 64% yield on our first attempt (Scheme 2a). The use of methyl *tert*-butyl ether (MTBE) to replace diethyl ether in the workup produced a yield of 71%. This method was repeated on a 500 g scale with NaOH as the base, and 73% yield was observed. We anticipate a significant reduction in the cost of acetone oxime prepared by this procedure compared to current commercial sources.

With a cheaper preparation of acetone oxime in hand, we also sought to improve the synthesis of oxime ester **9**. Our previous synthesis (Scheme 2b) used a slight excess of isobutyric acid chloride and triethylamine and 50 V of dichloromethane (DCM) as the solvent. We found that we could increase the throughput of the reaction by decreasing the solvent to 30 V, as well as increasing triethylamine addition from 1.1 to 1.2 equiv. These small changes afforded a 99% corrected yield on a 35 g scale. This reaction was also scaled up significantly, to 200 g, with a 90% corrected yield. No purification was necessary as the crude material was sufficient for use in the next step (vide infra). We hypothesize that this reaction could be further improved by replacing dichloromethane with a less hazardous solvent and that the reaction scale could be increased even further.

**Figure 2:**
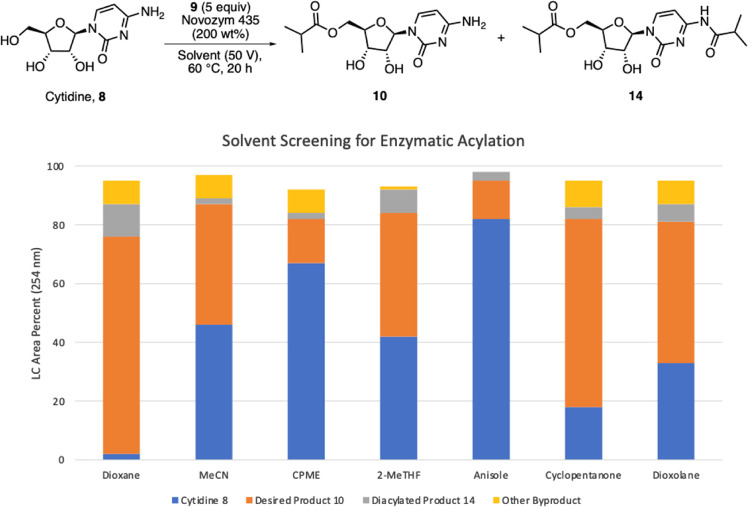
Various solvents screened for the enzymatic acylation of cytidine (MeCN = acetonitrile, CPME = cyclopentyl methyl ether, 2-MeTHF = 2-methyl tetrahydrofuran).

Streamlined preparation of acylating agent **9** facilitated investigation of the enzymatic acylation of cytidine (Figure 1, step 1). We first sought to investigate environmentally preferable solvents to substitute for 1,4-dioxane (dioxane).^7^ Unfortunately, extensive solvent screening (Figure 2, additional data in SI, Section 2.2) did not reveal any other solvent that worked as well as dioxane for the reaction.

Cyclopentanone appeared promising based on our initial screening, and its high boiling point (131 °C) opened the possibility for an increased reaction temperature that we hoped would accelerate conversion. Unfortunately, higher temperatures led to lower conversion (SI, Section 2.3), possibly due to the release of the enzymes from the polymer beads^8^ or degradation of the product. The lower conversion with cyclopentanone led us to return to dioxane as the optimal solvent. Finally, we did a cursory investigation to ascertain whether a lower temperature or concentration could benefit the reaction; we found that lowering the temperature to 40 °C lowered conversion (SI, Section 2.4). We then screened other enzymes in dioxane and additional solvents, but the originally identified Novozym-435 (N435) was the most efficient and selective enzyme for the desired 5′-*O*-acylation (SI, Section 2.5). It was determined that a lower stoichiometry of the acylating agent (4.0 vs 5.0 equiv) could be used (SI, Section 2.6); while the yield decreased slightly, we believe the savings in cost and purity offset the decrease in the yield. Thus, 4.0 equiv of oxime ester and 60 °C were selected as the optimal conditions.

**Figure 3:**
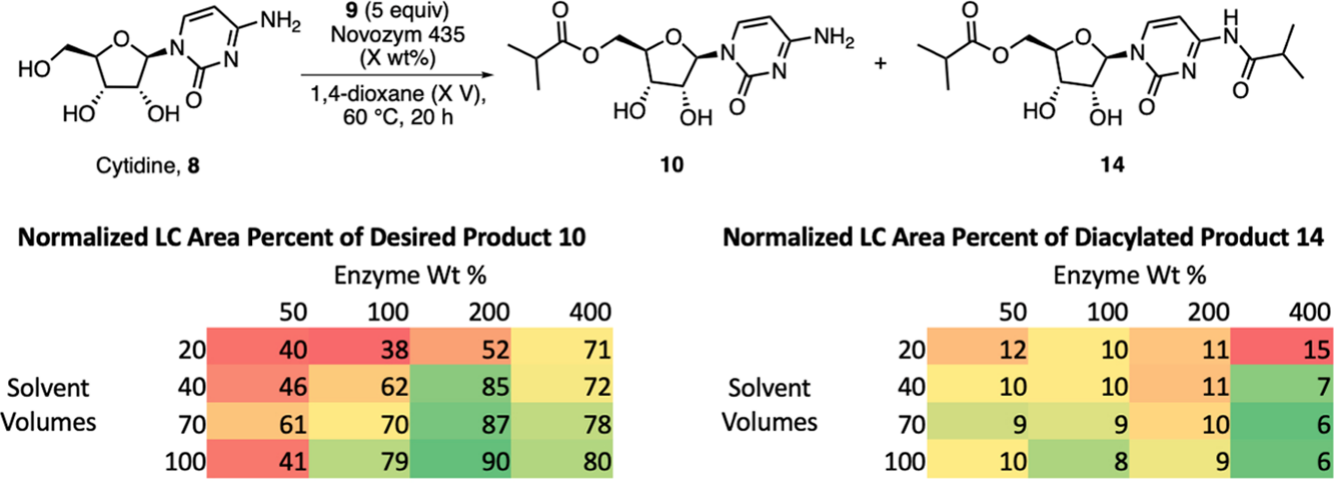
Assessment of solvent volume versus enzyme loading. See SI, Section 2.7, for additional details.

Hoping to decrease further the amount of the solvent needed for the reaction, a two-factor, four-level full factorial screening of 20–100 solvent volumes and 50–400 weight percent enzyme loading were performed (Figure 3).

We hoped that increased enzyme loading would allow us to decrease solvent usage, which would in turn improve the reaction throughput and process mass intensity (PMI). Unfortunately, our screening revealed that increasing concentration significantly hindered conversion; we hypothesize that this effect is due to the limited solubility of cytidine in dioxane. Ultimately, we chose conditions using 4.0 equiv of oxime ester, 150 wt % N435, and 50 V of dioxane as the solvent, due to the highest distribution of product **10** relative to remaining starting material **8** and diacylated product **14** (SI, Section 3.3).

Additionally, the breadth of data on regenerating and recycling N435, including repeated uses in organic solvents,^8,9a−9d^ suggests that N435 could be recycled for this reaction as well. Reuse of the enzyme would further decrease the raw material costs associated with this route.

Upon scaling up the reaction, we noticed a slowed reaction rate, from 24 h to completion at a small scale to approximately 40 h at a 19 g scale (SI, Section 2.8). We believe that this change is related to differences in the stirring method used at different scales: we stirred using magnetic stir bars at 750 rpm for scales up to 1 g, while larger-scale reactions used an overhead stirrer set to 260 rpm. Using this same setup, the reaction was scaled 10-fold to 200 g with no change in performance.

Throughout the reaction optimization, the major impurity observed was the diacylated product (**14**), the structure of which was confirmed as the N-acylated product via ^15^N–^1^H 2-D NMR spectroscopy (SI, Section 2.9). However, we also saw a consistent unknown impurity in the high-performance liquid chromatography (HPLC), constituting 5–10 LC area percent (SI, Section 2.10). We noticed that this impurity increased in proportion with enzyme loading, leading us to believe that the impurity was leached from the enzyme beads by dioxane. Indeed, a control reaction confirmed that the impurity formed simply from stirring the enzyme beads in dioxane at 60 °C overnight and was not related to either the cytidine or the acylating agent (SI, Section 2.9). Isolation of this impurity yielded a yellow gel that did not ionize on liquid chromatography-mass spectrometry (LC-MS). We hypothesize that this material is a macromolecular compound leached from the Lewatit VP OC 1600 solid support; solubilization of the support in studies of N435 catalysis has been reported for some solvents.^8^ Fortunately, simply rinsing the enzymes with dioxane before use eliminated this impurity (SI, Section 2.9). Thus, the main impurities remaining in the reaction were the diacylated product **14**, unreacted cytidine **8**, and excess acylating agent **9**. Allowing this reaction to cool to room temperature (RT, 20 °C) and then filtering enabled the removal of the enzyme beads as well as the unreacted starting material (SI, Section 3.2); this was due to cytidine’s insolubility in room-temperature dioxane.

The main challenge for purification was therefore removing the excess acylating agent and the diacylated product from the desired product **10**. Upon completion of the reaction, the remaining cytidine starting material as well as the enzyme beads were filtered out and then dioxane was removed via rotary evaporation. We then determined three methods (A, B, and C) for the purification of **10** from compounds **14** and **9** (Table 1). Methods A and B were successful for the purification of desired product **3** up to a purity of >99% on smaller scales (10–100 g), while purification C was successful on up to 200 g scale.

**Table 1:**
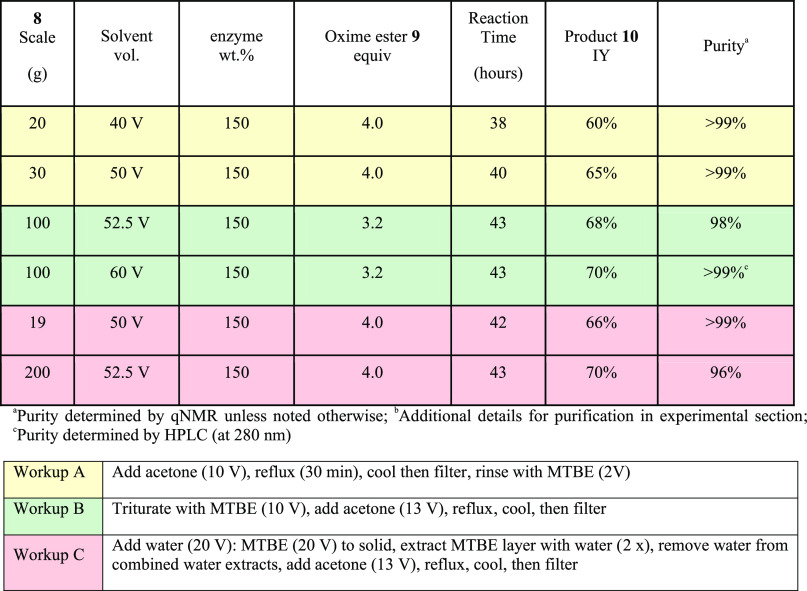
Cytidine Acylation Summary

The previous report of the transamination of intermediate **10**, although high yielding, was obtained using column chromatography, which would not be viable on the scale.^5^ Previously, 70% aq isopropanol was used as the solvent for this reaction; however, we chose to replace this with 70% aq 1-butanol solution. We hypothesized that the decreased solubility of butanol in water (as compared to the solubility of isopropanol in water) would decrease the amount of hydroxylamine salts present in the organic layer after reaction, thus leading to a more facile purification. The optimization of this transamination reaction began by using 4.5 equiv of hydroxylamine sulfate relative to the starting material **10**,^5^ which displayed a good conversion of **10** to the desired product **7** after 22 h (SI, Section 4.1). In addition to the desired product formation, the side product *N*-hydroxycytidine (NHC, **15**) was formed in this reaction in small quantities. We found that decreasing the stoichiometry of hydroxylamine sulfate from 4.5 equiv to 2 or 3 equiv led to very similar reaction conversion (Table 2 and SI, Section 4.1) and increased the purity of the crude reaction mixture after workup, which simply involved separating the organic and aqueous layers and then removing the 1-butanol from the organic layer (crude purity, Table 2).

**Table 2:**
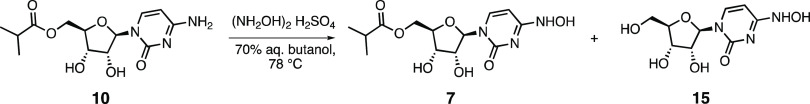
Development of Conditions for the Transamination of Compound **10** to Molnupiravir (**7**)

entry	scale of **10** (g)	(NH_2_OH)_2_ H_2_SO_4_ equiv	time (h)	**7** crude purity (%)^a^	**10** LC area %^b^	**7** LC area %^b^	**15** LC area %^b^
1	1	4.5	22	73	9	89	2
2	1	3	22	85	8	90	2
3	1	2	22	84	9	89	2
4	5	3	22	97	6	92	2

a Purity determined by qNMR.

b Determined by LC-MS at 280 nm.

After the development of successful small-scale conditions (5 g), the next goals were to scale up this reaction to 80 g and to develop recrystallization conditions for the crude reaction mixture. We found that increasing the scale from 5 to 10 to 20 g did not significantly affect the reaction time. However, increasing the scale to 80 g required an increased reaction time to 40 h for a similar conversion (Table 3).

**Table 3:** Scale-Up of the Transamination of **10** to **7** as well as the Isolated Yields and Purity of the Isolated Material

entry	scale of **10** (g)	(NH_2_OH)_2_ H_2_SO_4_ equiv	time (h)	**7** crude purity (%)^a^	**10** LC area %^a^	**7** LC area %^a^	**15** LC area %^a^	**7** isolated yield (%)	purity of isolated **7** (%)^b^
1	10	3	24	92	4	94	2	48	>99
2	20	3	28	93	2	96	2	49	>99
3	80	3.2	40	80	4	91	2	58	97

a Based on LC-MS area percent at 280 nm.

b Purity determined by qNMR.

We found that the precrystallization purification of this reaction on a 80 g scale worked the same as on a 1 g scale, by simply separating the aqueous and organic layers. To increase the purity of the final compound from 80 to 90% after this initial purification, recrystallization conditions were developed. Due to the difference in solubility of compound **7** in cold versus hot water (Table 4), water was chosen as the recrystallization solvent. Thus, it was determined that after the solvent was removed from the organic layer, **7** could be recrystallized in greater than 99% purity from heating to 65 °C and slowly cooling in water (2 V). After the initial recrystallization, the remainder of **7** remained in the aqueous filtrate. We anticipate that further recrystallizations of the filtrate would produce additional molnupiravir.

**Table 4:**
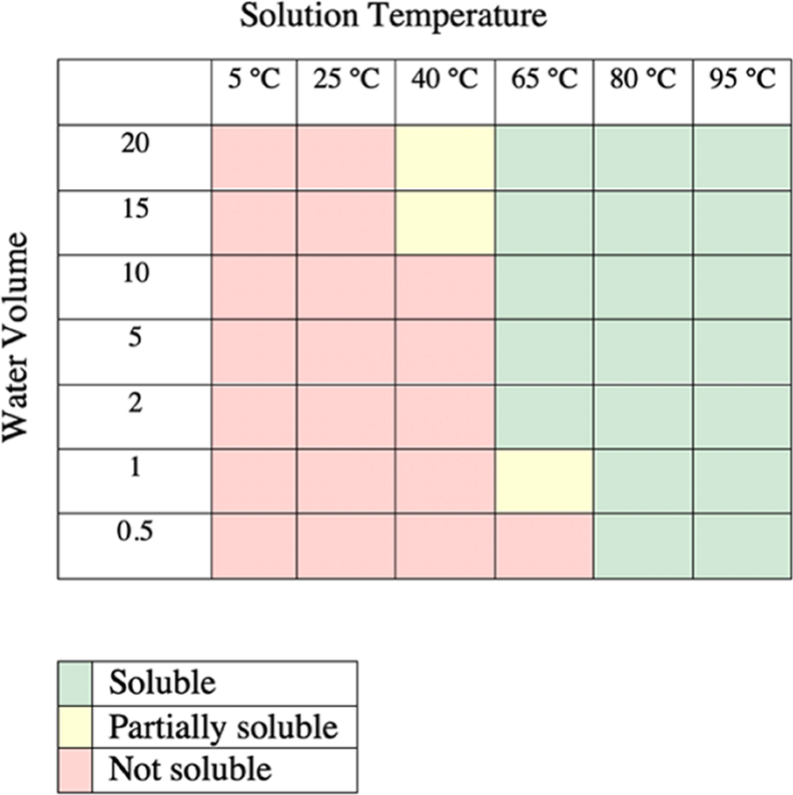
Qualitative Solubility Studies of Molnupiravir in Water at Various Solvent Volumes and Temperatures

## Conclusions

In conclusion, we have developed an increased scale, chromatography-free, two-step route from cytidine to molnupiravir with an overall isolated yield of 41% and lower cost as compared to the originally patented route^3^ (SI, Section 1), with further cost decreases possible if solvent and enzyme recycling is employed. We hope that further scale-up of this route will enable affordable, efficient preparation of this potentially crucial pharmaceutical for the global fight against COVID-19.

## Methods

### General Procedures

For all compounds, ^1^H and ^13^C NMR spectra were recorded on Bruker Avance III spectrometers (400, 500, or 600 MHz). Chemical shifts were measured relative to the residual solvent resonance for ^1^H and ^13^C NMR (CDCl_3_ = 7.26 ppm for ^1^H and 77.2 ppm for ^13^C, DMSO-*d*_6_ = 2.50 ppm for ^1^H, and 39.2 ppm for ^13^C). Coupling constants *J* are reported in hertz (Hz). The following abbreviations were used to designate signal multiplicity: s, singlet; d, doublet; t, triplet; hept, heptet; dd, doublet of doublet; and m, multiplet. Reactions were monitored by HPLC (Agilent 1260 Infinity II LC) or LC-MS (Agilent Technologies InfinityLab LC/MSD). Unless noted otherwise, reactions involving air-sensitive reagents and/or requiring anhydrous conditions were performed under a nitrogen or argon atmosphere with glassware oven-dried at 140 °C. Reactions requiring mechanical stirring were stirred using a Heidolph RZR 2020 overhead stirring apparatus. Purity was assessed by quantitative NMR (qNMR) spectroscopy with benzyl benzoate or dimethyl sulfone (Sigma-Aldrich TraceCERT grade) as the reference standard. Reagents and solvents were purchased from Aldrich Chemical Company, Fisher Scientific, Alfa Aesar, Acros Organics, Oakwood, or TCI. Unless otherwise noted, solid reagents were used without further purification. Methylene chloride (DCM) and dioxane were taken from a solid-sorbant Solvent Dispensing System purchased from Pure Process Technologies. Other solvents were purchased in anhydrous grades and used as received.

### Acetone Oxime (**11**)

#### 11 g Scale

To a solution of acetone (11.0 mL, 150 mmol, 1.0 equiv) and hydroxylamine hydrochloride (15.6 g, 225 mmol, 1.5 equiv) in HPLC-grade H_2_O (300 mL) at RT was added Na_2_CO_3_ (28.6 g, 270 mmol, 1.8 equiv) in portions. The reaction mass was stirred for 19 h at RT, after which it was extracted with Et_2_O (5 × 80 mL). The combined organic extracts were washed with saturated brine (40 mL) and dried over MgSO_4_. After filtration and solvent removal, the product was obtained as a white solid in 64% yield (7.051 g) and 98% purity (qNMR). ^1^H NMR (400 MHz, CDCl_3_) δ 8.33 (s, 1H), 1.90 (s, 3H), and 1.89 (s, 3H). ^1^H and ^13^C NMR matched reported literature values.^6^ Repetition of the reaction with MTBE instead of Et_2_O in the workup improved the yield to 71% (7.735 g).

#### 500 g Scale

To a solution of hydroxylamine hydrochloride (500 g, 7.20 mol, 1.1 equiv) in H_2_O (670 mL) at 0–5 °C was added a solution of NaOH (270 g, 6.75 mol, 1 equiv) in H_2_O (670 mL) dropwise. The reaction mass was stirred for 30 min at 0–5 °C and acetone (500 mL, 6.75 mol, 1 equiv) was added. The reaction mass was stirred for a further 2 h at 0–5 °C, after which it was filtered and vacuum-dried for 45 min yielding 450 g of wet white solid. The solid was then dissolved in DCM (9.0 L, 20 V) and stirred for 15 min, after which the layers were separated. The DCM layer was dried with Na_2_SO_4_ and washed with DCM (2.25 L, 5 V), then dried, yielding a white solid in 73% yield (360 g). The material was carried forward without further purification.

### Acetone Oxime *O*-Isobutyryl Ester (**9**)

#### 35 g Scale

Acetone oxime (35.0 g, 478.9 mmol, 1.0 equiv) was dissolved in dichloromethane (1050 mL, 30 V) under an argon atmosphere with mechanical stirring and cooled to 0 °C using an ice/water bath. Isobutyryl chloride (55.2 mL, 526.7 mmol, 1.1 equiv) was slowly added, maintaining the solution temperature between 0 and 5 °C. Et_3_N (31.46 mL, 574.7 mmol, 1.2 equiv) was added via a syringe pump at a rate of 2 mL/min, again maintaining the solution temperature below 5 °C. Et_3_N addition caused the evolution of vapor and the formation of solid precipitates. The reaction mass was stirred for 20 h and allowed to warm to room temperature. The reaction mass was then washed with H_2_O (2 × 350 mL), 5% w/w solution of NaHCO_3_ (2 × 250 mL), H_2_O (1 × 350 mL), 1 N aq HCl (2 × 250 mL), H_2_O (1 × 350 mL), and saturated brine solution (1 × 100 mL), followed by drying over MgSO_4_. After filtration and solvent removal, the product was obtained as a pale yellow liquid in 68.81 g yield (99% corrected for purity) and 99% purity (qNMR, benzyl benzoate). The material was carried forward without purification. ^1^H NMR (400 MHz, CDCl_3_) δ 2.66 (hept, *J* = 7.0 Hz, 1H), 2.05 (s, 3H), 1.99 (s, 3H), 1.24 (d, *J* = 7.0 Hz, 6H). ^1^H and ^13^C NMR matched previously reported values.^5^

#### 200 g Scale

Acetone oxime (200 g, 2.74 mol, 1.0 equiv) was dissolved in DCM (6 L) and cooled to 0 °C using an ice/water bath. Isobutyryl chloride (325 mL, 3.01 mol, 1.1 equiv) and Et_3_N (241 mL, 3.28 mol, 1.2 equiv) were slowly added, maintaining the solution temperature between 0 and 5 °C. The reaction mass was stirred for 20 h and allowed to warm to room temperature. The reaction mass was then washed with H_2_O (2 × 2 L), 5% w/w solution of NaHCO_3_ (2 × 1.4 L), H_2_O (1 × 1.4 L), 1 N aq HCl (2 × 1.4 L), H_2_O (1 × 2 L), and saturated brine solution (1 × 600 mL), followed by drying over Na_2_SO_4_. After filtration and solvent removal, the product was obtained as a pale yellow liquid in 385 g yield (90% corrected for purity) and 92% purity (qNMR). The material was carried forward without purification.

### 5′-*O*-Isobutyryl Cytidine (**10**)

#### 30 g Scale

Novozym-435 (45.0 g) was rinsed with 1,4-dioxane (2 × 100 mL) in a sintered glass funnel and dried under vacuum for 20 min. To an oven-dried 3 L three-neck RBF were added cytidine (30.0 g, 123.3 mmol), rinsed enzyme beads, 1,4-dioxane (1.5 L, 50 V), and crude acetone oxime *O*-isobutyryl ester (70.65 g, 493.4 mmol). The reaction mixture was heated to 64 °C using a heating mantle and stirred using a mechanical stirrer for 39 h, monitoring by HPLC (see SI for HPLC traces). The reaction mixture was then cooled to room temperature and filtered and washed with dioxane (3 V). The solvent was removed by rotary evaporation, yielding the crude product as an off-white foam (the crude material for this reaction was then purified via purification method A, see SI, Section 3.1, for other purification procedures).

Purification A: Acetone (300 mL, 10 V) was added to the solid and then refluxed for 30 min. The suspension was allowed to cool to room temperature and then cooled to 5 °C (12 h). The white solid was filtered and rinsed with MTBE (2 V) and then allowed to dry at 40 °C (12 h) under vacuum to yield compound **10** (25.2 g, 65%, >99% purity by qNMR). ^1^H NMR (500 MHz, DMSO) δ 7.58 (d, *J* = 7.4 Hz, 1H), 7.19 (d, *J* = 23.1 Hz, 2H), 5.76 (d, *J* = 3.7 Hz, 1H), 5.73 (d, *J* = 7.4 Hz, 1H), 5.40 (d, *J* = 5.2 Hz, 1H), 5.19 (d, *J* = 5.9 Hz, 1H), 4.28 (dd, *J* = 12.2, 3.2 Hz, 1H), 4.18 (dd, *J* = 12.1, 5.4 Hz, 1H), 3.99–3.94 (m, 2H), 3.93–3.87 (m, 1H), 2.58 (hept, *J* = 7.0 Hz, 1H), 1.11 (d, *J* = 7.0 Hz, 6H). ^1^H and ^13^C NMR matched the previously reported values.^5^

#### 200 g Scale

Novozym-435 (300 g, 150 wt %) was rinsed with 1,4-dioxane (4 L) and dried under vacuum for 30 min. Cytidine (200 g, 822 mmol, 1 equiv), rinsed enzyme beads, 1,4-dioxane (10.5 L, 52.5 V), and crude acetone oxime *O*-isobutyryl ester (471 g, 3.29 mol, 4 equiv). The reaction mixture was heated to 60 °C and stirred for 43 h. The reaction mixture was then cooled to room temperature, filtered, and washed with dioxane (3 V). The solvent was removed by distillation.

Purification C: MTBE (20 V) and H_2_O (20 V) were then added. The layers were separated and the organic layer was washed twice with H_2_O. The combined aqueous layers were distilled to yield a white solid. Acetone (13 V) was added and the slurry was heated to 60 °C for 1 h, cooled to room temperature, filtered, and dried, yielding 188 g (70% corrected) with a purity of 96.5% (qNMR).

### Molnupiravir (**7**)

#### 10 g Scale

5′-*O*-Isobutyryl cytidine (10.0 g, 32.0 mmol, 1.0 equiv) was dissolved in 70% aq 1-butanol (112 mL of 1-butanol: 48 mL of water) and then hydroxylamine sulfate (15.8 g, 96.0 mmol, 3.0 equiv) was added. The mixture was stirred vigorously and heated to 78 °C for 24 h. The layers were then separated, and 1-butanol was removed from the organic layer via rotary evaporation to yield the solid white, crude material (5.2 g, 92% purity via qNMR). This crude material was then dissolved in water (2 V) and heated to 65 °C for 30 min (60 min for 80 g scale). After completely dissolved, the mixture was allowed to cool to room temperature and then to 5 °C without stirring. The solid was then filtered and washed with MTBE (2 V) to obtain molnupiravir (5.03 g, 48% yield, >99% purity by qNMR). ^1^H NMR (500 MHz, DMSO) δ 10.00 (s, 1H), 9.53 (s, 1H), 6.83 (d, *J* = 8.2 Hz, 1H), 5.72 (d, *J* = 5.5 Hz, 1H), 5.59 (dd, *J* = 8.2, 2.0 Hz, 1H), 5.36 (d, *J* = 5.6 Hz, 1H), 5.21 (s, 1H), 4.21 (dd, *J* = 12.0, 3.3 Hz, 1H), 4.14 (dd, *J* = 12.0, 5.1 Hz, 1H), 4.02–3.97 (m, 1H), 3.94–3.88 (m, 2H), 2.58 (hept, *J* = 7.0 Hz, 1H), 1.11 (dd, *J* = 7.0, 1.0 Hz, 6H). ^1^H and ^13^C NMR matched the previously reported values.^5^

#### 80 g Scale

5′-*O*-Isobutyryl cytidine (80 g, 255 mmol, 1.0 equiv) was dissolved in 70% aq 1-butanol (20 V) and then hydroxylamine sulfate (134 g, 817 mmol, 3.2 equiv) was added. The mixture was stirred vigorously and heated to 75–80 °C for 40 h. The mixture was cooled to room temperature, the layers were then separated, and 1-butanol was distilled from the organic layer yielding a solid white, crude material (85 g). This crude material was then dissolved in water (2 V) and heated to 60–65 °C for 60 min. After completely dissolved, the mixture was allowed to cool to room temperature and stirred for 1 h and then cooled to 5–10 °C and stirred for 3 h. The solid was then filtered and wet-washed with MTBE (3 V) to obtain 56.0 g of molnupiravir. The purification was repeated again for a final yield of 50.0 g (58% corrected) with 97% purity (qNMR).

## Supporting Information Available

Route cost calculations, additional experimental details for the transformations of compounds **8** to **10** and **10** to **7**, NMR data for compounds **14**, **11**, **9**, **10**, and **7** (PDF)
