# Supplementary material for: Progress Toward a Large-Scale Synthesis of Molnupiravir (MK-4482, EIDD-2801) from Cytidine
Source: ACS Omega. 2021 Apr 8;6(15):10396–402. doi: 10.1021/acsomega.1c00772 (PMC8153789; doi:10.1021/acsomega.1c00772)
Supplement: Supplementary file 1 — ao1c00772_si_001.pdf [file ao1c00772_si_001.pdf]

Supplementary Information for:

Progress Towards a Large-Scale Synthesis of Molnupiravir (MK-4482, EIDD-2801) from Cytidine

Grace P. Ahlqvist,[1] Catherine P. McGeough,[1] Chris Senanayake,[2] Joseph D. Armstrong,[2] Ajay Yadaw,[2] Sarabindu Roy,[2] Saeed Ahmad,[3] David R. Snead,[3] Timothy F. Jamison,[1,]*

1 Department of Chemistry, Massachusetts Institute of Technology, 77 Massachusetts Ave, Cambridge, MA 02139, United States

2 TCG GreenChem, Inc., Process R&D Center at Princeton South, 701 Charles Ewing Boulevard

Ewing, NJ 08628, United States

3 Medicines for All Institute, 737 N. 5th St., Box 980100, Richmond, VA, United States

Contents

1. Technoeconomic Analysis of MK-4482 Synthesis……………………………………………………………S3

2. Enzymatic Acylation of Cytidine **8**
    1. Acyl Donor Screening……………………………………………………………………………………S4
    2. Solvent Screening…………………………………………………………………………………………S5
    3. Cyclopentanone Temperature Screening……………………………………………………………S6
    4. Dioxane Temperature Screening………………………………………………………………………S7
    5. Enzyme Screening…………………………………………………………………………………………S8
    6. Oxime Ester Stoichiometry……………………………………………………………………………..S10
    7. Solvent Volume and Enzyme Loading Screening……………………………………………..S11
    8. Timepoint Data……………………………………………………………………………………………..S12
    9. Identification of Diacylated Impurity **14**…………………………………………………………S13
    10. Determination of Enzyme Leaching Impurity………………………………………………….S15

3. Purification of 5′-*O*-isobutyrylcytidine **10**
    1. General Purification Procedures for Compound 10………………………………………….S16
    2. Additional Filtration Information………………………………………………………………….S16
    3. Additional Purification Information……………………………………………………………...S17

4. Transamination of 5′-*O*-isobutyrylcytidine **10**
    1. Optimization of Transamination Reaction……………………………………………………….S18

5. NMR Spectra
    1. ^{1}H NMR of Compound **11**……………………………………………………………………….S19
    2. ^{1}H NMR of Compound **9**………………………………………………………………………….S20
    3. Quantitative ^{1}H NMR of Compound **10**…………………………………………………….S21
    4. Quantitative ^{1}H NMR of Compound **7**……………………………………………………….S23

Section 1. Technoeconomic Analysis

EIDD-2801: Updated MIT Approach

Report 2/3/21 for larger scale run by TCG

RM Cost >10% of total (column "% RM Cost / Total RM cost")

Reaction Step:	Materials solvents to be	RM Cost $ / kg	Equivalent Volume	Amount kg	Amount / kg product	RM Cost $ / batch	RM cost $ / kg product	% RM Cost / Total RM cost	kg RM	kg RM / kg product	kg RM	kg RM / kg product
13 to 11:	**Acetone**	0.85	1.0	58.08	2.65	49.37	2.25	0.53%	58.08		58.08	
	Hydroxylamir	2.30	1.07	74.08	3.38	170.39	7.78	1.82%	74.08		74.08	
	Sodium hydro	0.48	1.0	80.01	3.65	38.40	1.75	0.41%	80.01		80.01	
	Water	0.06	3	143.12	6.53	8.59	0.39	0.09%	143.12			
	DCM	0.68	4	84.84	3.87	57.69	2.63	0.62%	84.84		84.84	
	Sodium sulfate	0.70	0.5	71.02	3.24	49.71	2.27	0.53%	71.02		71.02	
**73.0%**	**Product: Acetone oxime**			53.34	**2.44**	374.15	**7.01**	**4.00%**	**511.15**	**9.58**	**368.03**	**6.90**
11+12 to 9:	Isobutyryl chloride	1.10	1.1	117.21	3.91	128.93	4.30	1.01%	117.21		117.21	
	Acetone oxim	**7.01**	1.0	73.09	**2.44**	512.70	17.08		700.43		504.32	
	Triethylamine	2.06	1.2	121.43	4.05	250.14	8.33	1.95%	121.43		121.43	
	DCM	0.68	30	726.33	24.20	493.91	16.46	3.85%	726.33		726.33	
	Water	0.06	50	5,860.25	195.26	351.62	11.72	2.74%	5,860.25			
	5% NaHCO3 s	0.28	14	84.96	2.83	23.79	0.79	0.19%	84.96		84.96	
	Water for 5%	0.06		1,614.16	53.78	96.85	3.23	0.76%	1,614.16			
	1N HCl	0.75	14.3	66.74	2.22	50.05	1.67	0.39%	66.74		66.74	
	water for 1N H	0.06		1,787.11	59.54	107.23	3.57	0.84%	1,787.11			
	Saturated brine (25% NaCl)	0.40	3	92.48	3.08	36.99	1.23	0.29%	92.48		92.48	
	water for brine solution	0.06		277.43	9.24	16.65	0.55	0.13%	277.43			
	Sodium sulfate	0.70	0.5	58.60	1.95	41.02	1.37	0.32%	58.60		58.60	
**90.0%**	**Product of Step 2: oxime ester**			128.87	**4.29**	**2,109.87**	**16.37**	**12.46%**	**11,507.13**	**89.29**	**1,772.06**	**13.75**
8+9 to 10:	**Cytidine**	57	1.0	243.22	1.82	13,863.54	103.89	24.32%	243.22		243.22	
	CALB enzyme	50	*150%*	364.83	2.73	18,241.50	136.70	32.00%	364.83		364.83	
	**Oxime ester**	**16.37**	4.0	572.99	**4.29**	9,380.99	70.30		51,163.53		7,878.99	
	1,4 Dioxane	2.50	76	4,742.27	35.54	11,855.68	88.84	20.80%	4,742.27		4,742.27	
	H2O	0.06	30	7,296.60	54.68	437.80	3.28	0.77%	7,296.60			
	Methyl *tert*-b	1.07	20	900.40	6.75	963.43	7.22	1.69%	900.40		900.40	
	Acetone	0.85	13	620.12	4.65	527.10	3.95	0.92%	620.12		620.12	
**70.0%**	**Product of Step 3**			219.32	**1.64**	**55,270.04**	**252.01**	**80.50%**	**65,330.97**	**297.88**	**14,749.84**	**67.25**
**10 to 7:**	**Step 3 product**	**252.01**	1.0	313.91	**1.64**	79,108.41	414.18		93,508.69		21,111.55	
	(NH3OH)2.H2	2.59	3.2	525.25	2.75	1,360.39	7.12	1.67%	525.25		525.25	
	H2O	0.06	10	3,139.10	16.44	188.35	0.99	0.23%	3,139.10			
	1-Butanol	0.63	14	889.93	4.66	560.66	2.94	0.69%	889.93		889.93	
	Methyl tert-b	1.07	6	348.63	1.83	373.03	1.95	0.46%	348.63		348.63	
**58.0%**	**Product of Step 2 MK-4482 / EIDD-2801**			191.00	**1.00**	**81,590.84**	**427.18**	**3.04%**	**98,411.60**	**515.24**	**22,875.36**	**119.77**
						with solvent recycle	**427.18**	**100.00%**		**515.24**		**119.77**
	**Overall Yield**	**26.7%**				**1 Mole KRM case**	**$ / kg**	**Sum of**		**PMI**		**PMI**
						w/o solvent recycle	**799.15**	**step %s**		**all**		**without**
	**solvent recycle**	**75%**						**100.00%**		**RMs**		**water**
						with solvent recycle	***427.18***			***515.24***		***119.77***
			RM Cost & PMI from 1 kg Product Case				***$ / kg***					
						w/o solvent recycle	***799.15***			***759.72***		***364.24***

Figure S1. Calculations for the overall process cost per kg with and without 75% solvent recycling.

Section 2. Enzymatic Acylation of Cytidine

2.1 Acyl Donor Screening

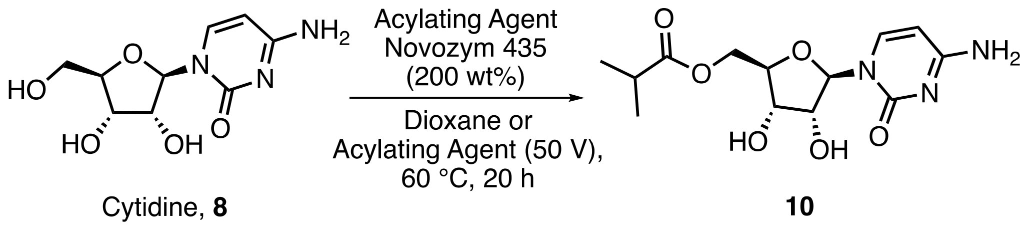

Entry	Acylating Agent	Amount	Result
1	Methyl Isobutyrate	5 equiv	No conversion
2		50 V (as solvent)	No conversion
3	Ethyl Isobutyrate	5 equiv	No conversion
4		50 V (as solvent)	No conversion
5	Isobutyric Anhydride	1.1 equiv	Over-acylation

Figure S2. Screening of methyl isobutyrate, ethyl isobutyrate, and isobutyric anhydride as potential acyl donors for cytidine.

Unfortunately, simple esters were not reactive enough to serve as acyl donors for this reaction. In addition, the uncatalyzed background reaction between cytidine and isobutyric anhydride was too rapid to suppress, yielding a complex mixture of products acylated at multiple positions. We therefore confirmed our choice of acetone oxime *O*-isobutyryl ester as a moderately activated acylating agent that is ideal for use in this reaction.

2.2 Solvent Screening

In addition to solvent screening data presented in the main text, we also screened many of the same solvents as well as additional solvents with similar conditions (except using 3 equiv oxime ester instead of the previously used 5 equiv).

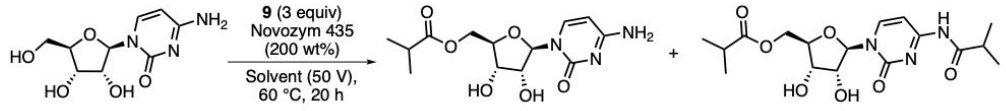

Cytidine, **8** **10** **14**

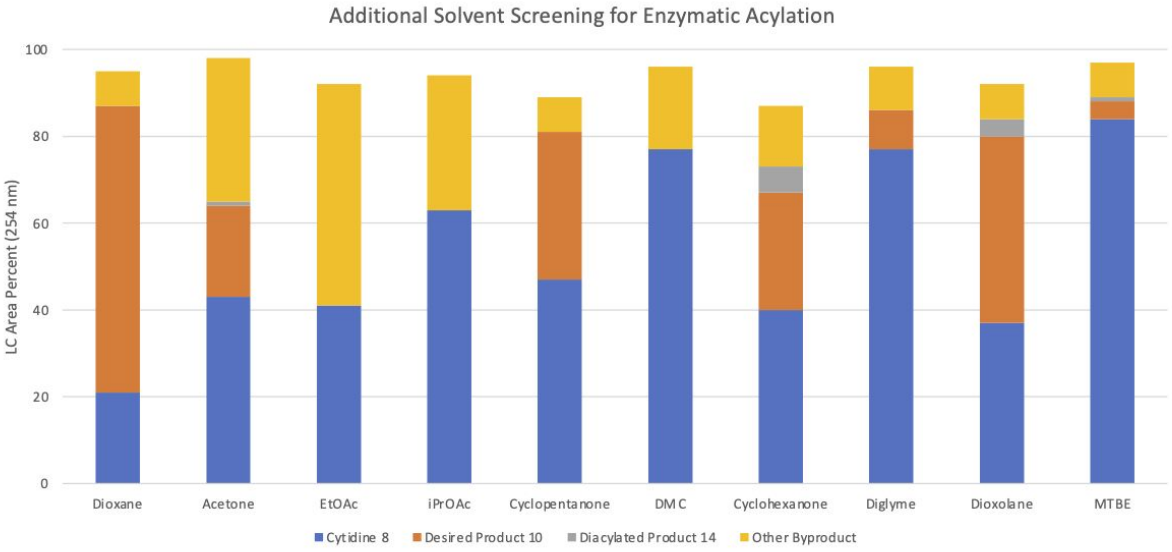

Figure S3. Solvent screen using 3 equivalents of the oxime ester.

We hoped that simple ester or alcohol solvents could be viable for the reaction due to the above results with methyl and ethyl isobutyrate; however, less hindered esters such as ethyl acetate led to significant amounts of byproducts that were not investigated further. Thus, we confirmed dioxane as the choice of solvent for the reaction.

2.3 Cyclopentanone Temperature Screening

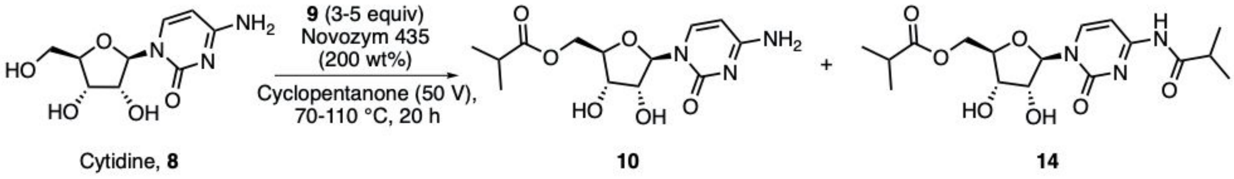

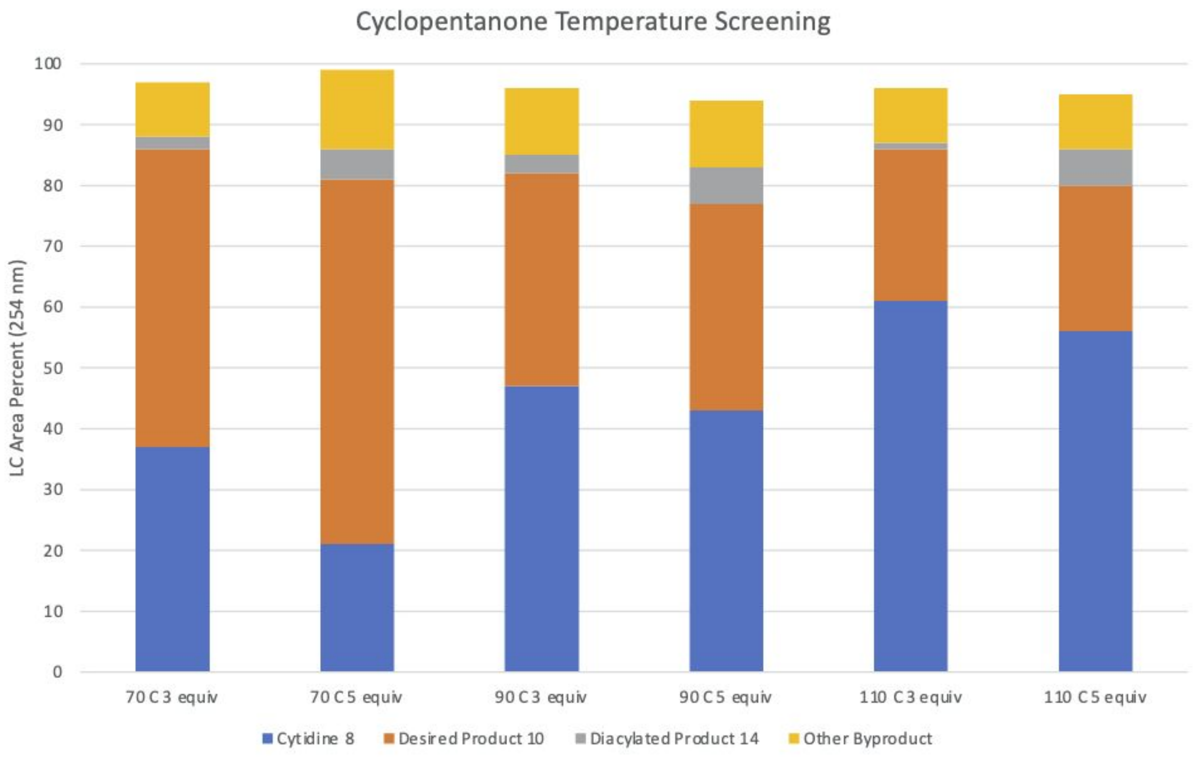

Figure S4. Reaction temperature screening with cyclopentanone as the solvent.

2.4 Dioxane Temperature Screening

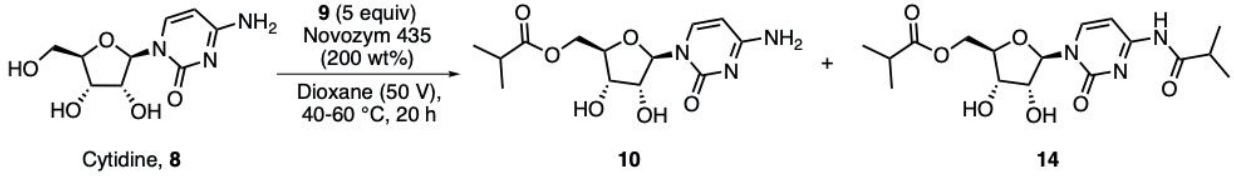

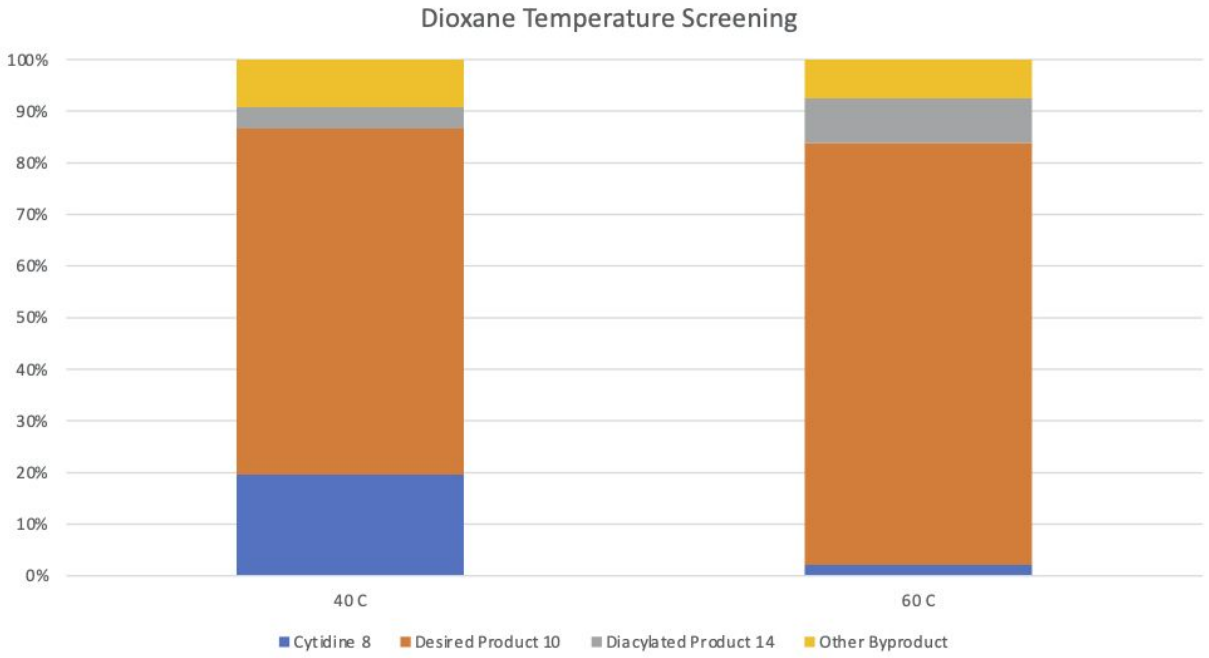

Figure S5. Reaction temperature screening with dioxane as the solvent.

2.5 Enzyme Screening

Additional enzymes were briefly screened to assess viability.

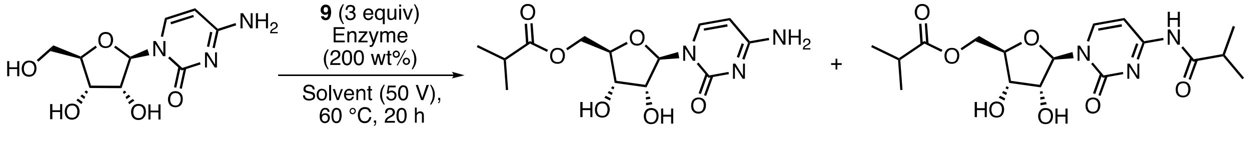

Entry	Enzyme	Vendor	Catalog Number	Desired Product 10 in Dioxane (LC Area %)	Desired Product 10 in 2-MeTHF (LC Area %)
1	Novozym-435	Sigma-Aldrich	L4777	66	22
2	CAL-B on Immobead 150	Sigma-Aldrich	54326	58	23
3	Lipozyme RM	Strem	06-3120	5	1
4	Candida rugosa lipase on Immobead 150	Sigma-Aldrich	89444	0*	0

*5% conversion to a compound with slightly different retention time (5.608 min vs. 5.748 min for desired product), possibly a different *O*-acylated compound

Figure S6. Screening of Novozym-435, CAL-B Immobead 150, Lipozyme RM, and Candida rugosa lipase on Immobead 150 as potential enzymes for selective acylation of cytidine.

Lipozyme RM (Entry 3) and *Candida rugosa* lipase on Immobead 150 (Entry 4) were not screened further. CAL-B on Immobead 150 showed some promise in initial screening, but further screening did not reveal conditions competitive to our initial results with dioxane and Novozym-435.

2.5 Enzyme Screening Continued

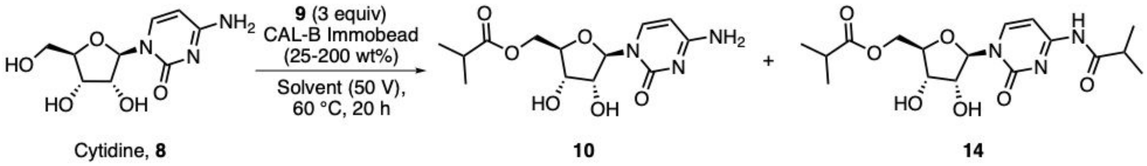

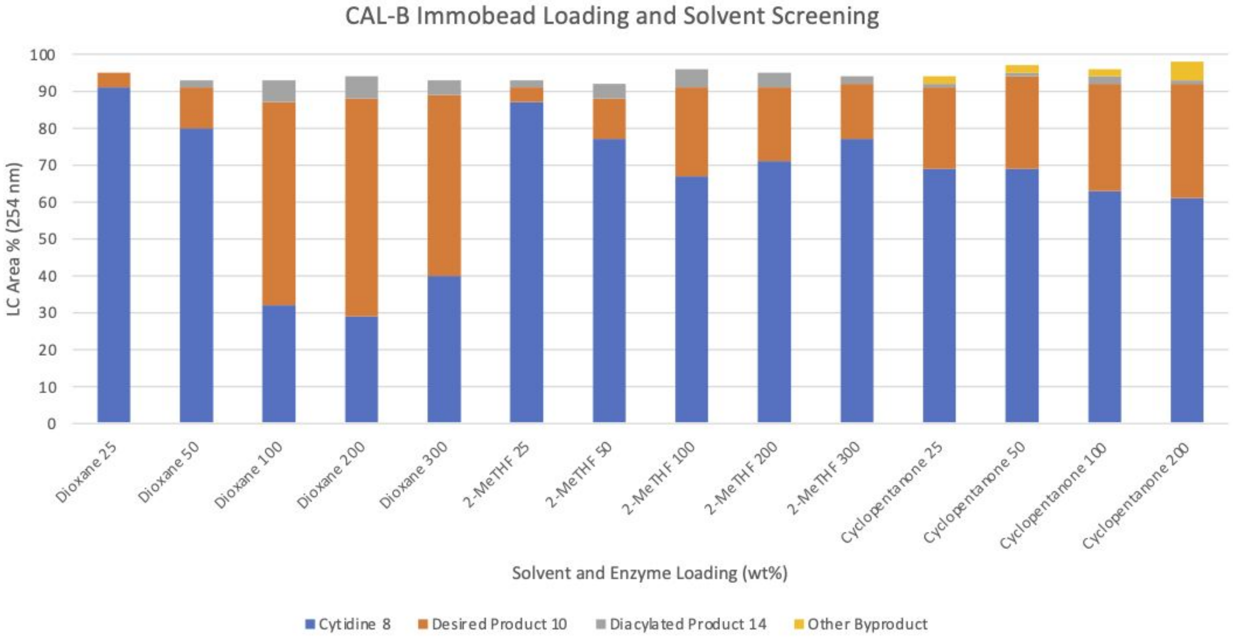

Figure S7. Additional acylation reaction screening conditions varying solvent and enzyme catalyst loading.

2.6 Oxime Ester Stoichiometry

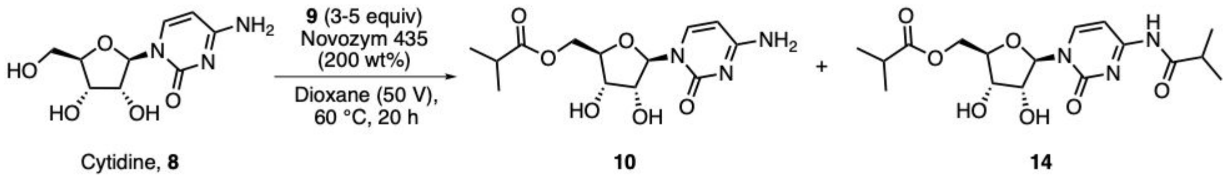

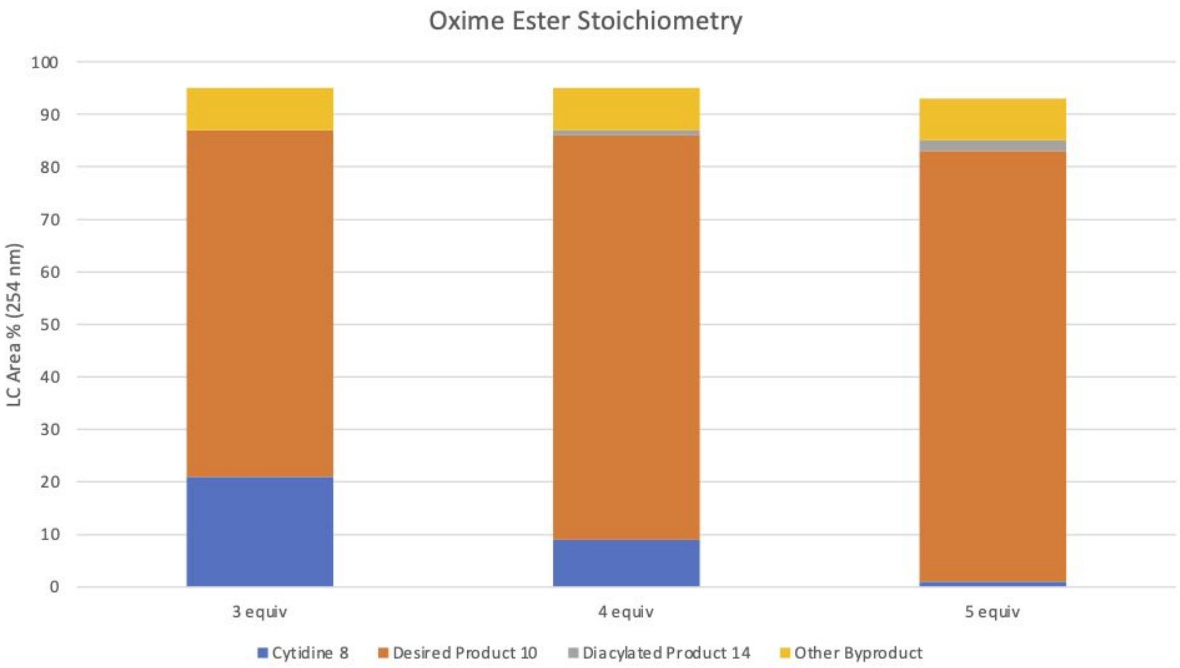

Figure S8. Reaction screenings varying the stoichiometry of acylating agent **9**.

Additional oxime ester increased conversion, but also increased formation of the diacylated side product. Ultimately, 4 equivalents of oxime ester was chosen as a compromise between conversion, cost, and purity.

2.7 Solvent Volume and Enzyme Loading Screening

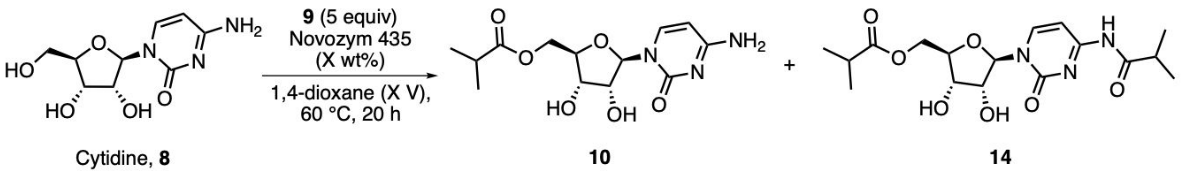

Normalized LC Area Percent of Desired Product 10

Solvent Volumes	Enzyme Wt % 50	100	200	400
20	40	38	52	71
40	46	62	85	72
70	61	70	87	78
100	41	79	90	80

Normalized LC Area Percent of Diacylated Product 14

Solvent Volumes	Enzyme Wt % 50	100	200	400
20	12	10	11	15
40	10	10	11	7
70	9	9	10	6
100	10	8	9	6

Figure S9. Assessing desired product (**10**) formation with varying solvent volumes and enzyme weight percentages (left), while also assessing diacylated side product (**14**) formation (right). (Main text Figure 3, reproduced here for convenience.)

The proportion of enzyme impurity (see SI Section 2.9) increased with the enzyme loading used, skewing the ratios across rows. Thus, we normalized the LC area percent (LCAPs) to better reflect the relevant ratios of **8** to **10** to **14** using the following equation:

$$Normalized\ LCAP\ of\ 10\ or\ 14 = \frac{LCAP\ of\ 10\ or\ 14}{LCAP\ of\ 8 + LCAP\ of\ 10 + LCAP\ of\ 14}$$

Colors in the chart for desired product **10** go from red at low LCAP and green at high LCAP. Colors for diacylated product **14** go from green at low LCAP to red at high LCAP.

2.8 Timepoint Data

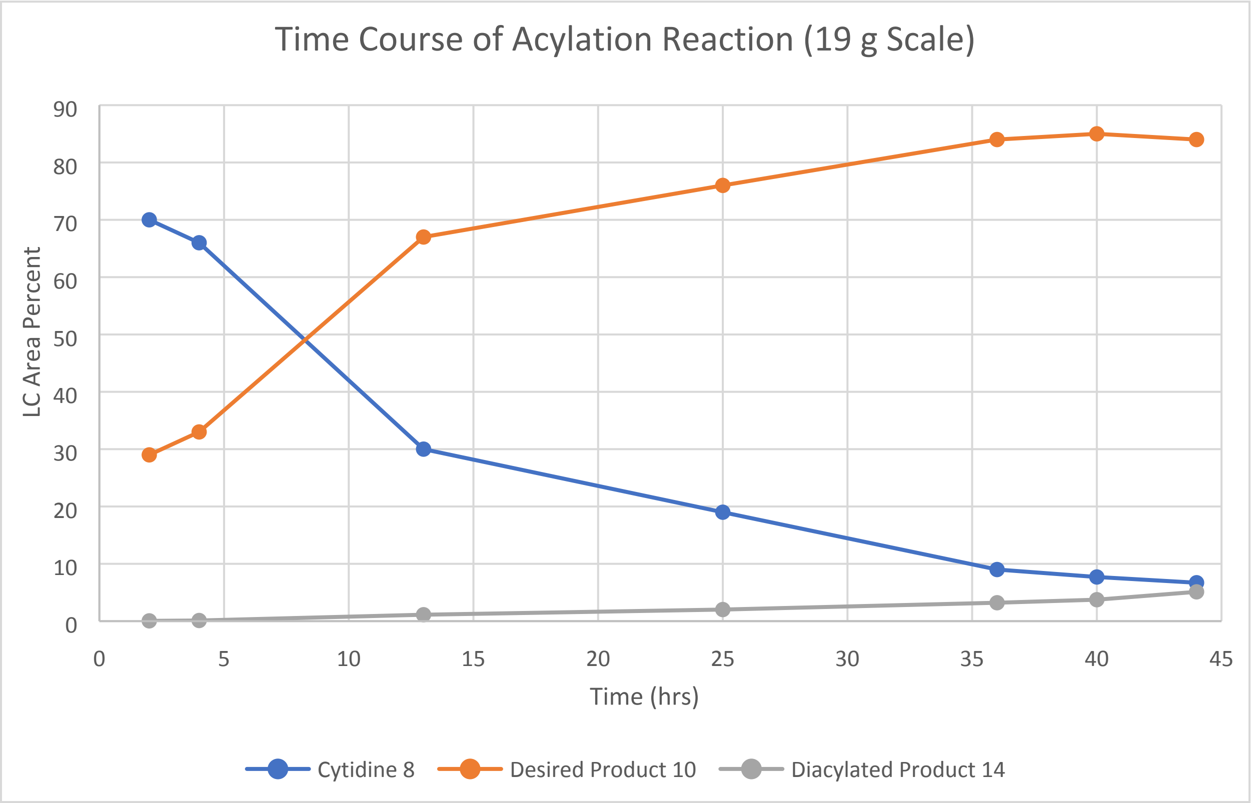

Figure S10. Product formation, starting material depletion, as well as diacylated material formation over 45 hours for a 19 g scale acylation reaction.

2.9 Identification of Diacylated Impurity

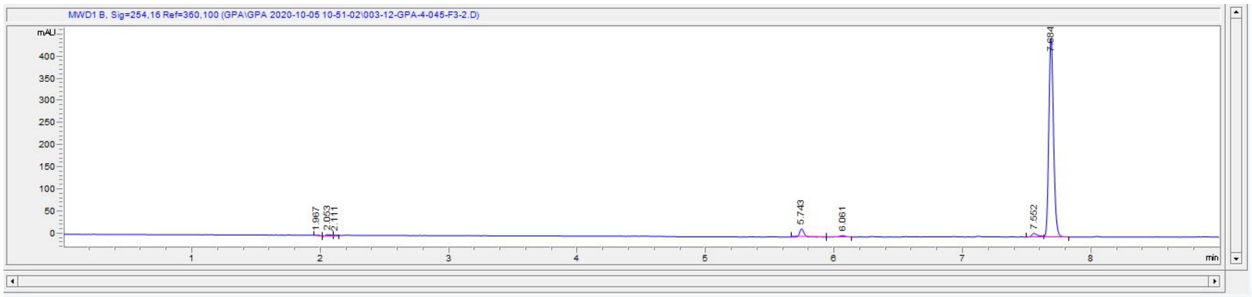

File Information

LC-File	003-12-GPA-4-045-F3-2.D
File Path	C:\Users\Public\Documents\ChemStation\1\Data\GPA\GPA 2020-10
Date	05-Oct-20, 11:23:36
Sample	GPA-4-045-F3-2
Sample Info	
Barcode	
Operator	SYSTEM
Method	Zorbax-Eclipse-Plus-C18_250mm_9min_10-70.M
Reference	C:\Users\Public\Documents\ChemStation\1\Data\GPA\GPA 2020-10

#	Time	Type	Area	Height	Width	Area%	Symmetry
1	1.967	VB	6.7	2	0.0441	0.533	0.392
2	2.053	BV	12.9	3.8	0.0499	1.027	0.53
3	2.111	VV	5.4	2.1	0.0342	0.432	0.292
4	5.743	BV R	45	18.7	0.037	3.573	0.747
5	6.061	VB R	11	3.2	0.0506	0.876	1.132
6	7.552	BV E	28	8.5	0.0486	2.228	0.554
7	7.684	VB R	1149.6	452.6	0.0396	91.330	0.927

Figure S11. Impurity isolated from crude acylation product by column chromatography (Biotage Isolera, DCM/methanol gradient) as a yellow oil. The Rf of the isolated product was confirmed by LC as the impurity of interest. Its mass was identified as 384.2 g/mol (HPLC-MS, $[M+H^+]$), corresponding to the mass of desired product **10** plus one additional isobutyryl ester unit. ^{15}N-^{1}H HSQC NMR spectrums supports the assignment of this impurity as the *N*-acylated constitutional isomer of the diacylated product.

2.9 Identification of Diacylated Impurity Continued

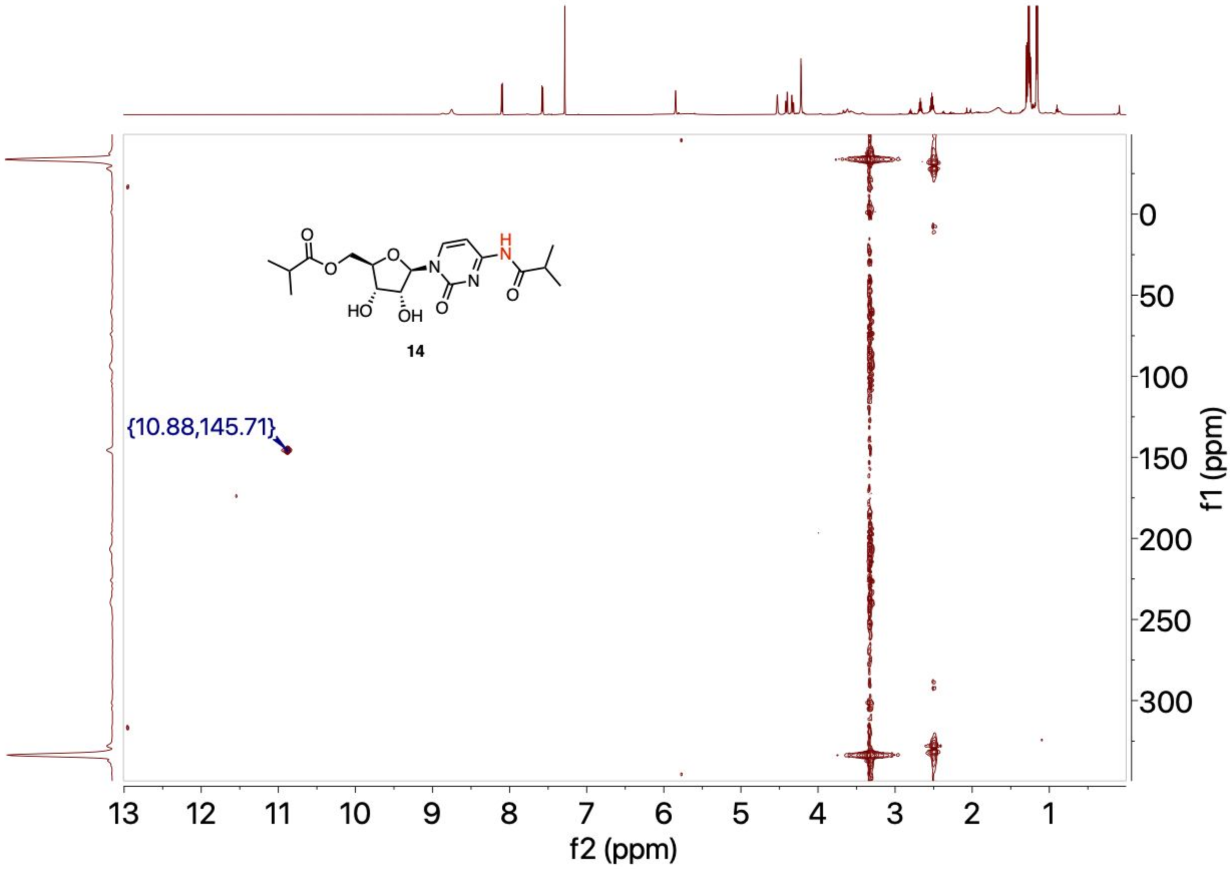

Figure S12. ^{15}N-^{1}H HSQC NMR spectrum of compound **14** with identified N-H correlation highlighted in red (600 MHz, DMSO-d_6).

2.10 Determination of Enzyme Leaching Impurity

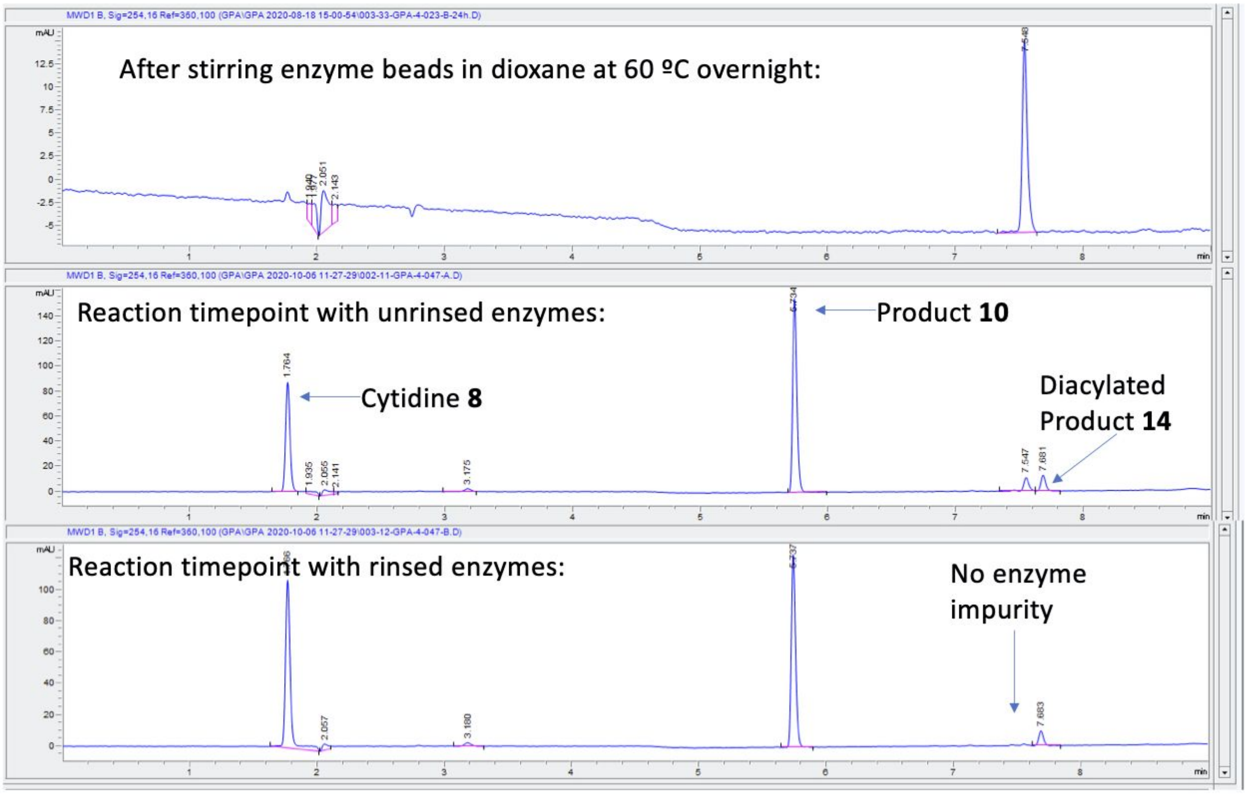

Figure S13. HPLC trace of dioxane after stirring with enzyme beads overnight at 60 °C (top); HPLC trace of reaction with unrinsed enzyme beads (middle); HPLC trace of reaction with rinsed enzyme beads (bottom).

Since the impurity at 7.55 min is observed in the control reaction with just enzyme beads, we concluded that it was arising from the beads and not the reaction itself. An HPLC of the reaction run with enzymes rinsed with dioxane before use shows that a simple rinse removes the impurity.

Section 3: Purification of 5'-*O*-isobutyrylcytidine 10

3.1 General Purification Procedures for Compound 10

Purification A: Acetone (10 V) was added to solid then refluxed for 30 minutes. The suspension was allowed to cool to room temperature then cooled to 5 °C (12 hours). The white solid was then filtered and rinsed with MTBE (2 V) then allowed to dry at 40 °C (12 h) under vacuum to yield compound **10.**

Purification B: The crude, off-white foam was triturated with MTBE (10 V). Acetone (13 V) was added to the solids, which were then refluxed for 3 hr. The suspension was allowed to cool to room temperature and then was filtered to yield white solid **10**.

Purification C: Water (20 V) and MTBE (20 V) were added to the off-white foam. The layers were then separated, and the MTBE layer was extracted with water (20 V, 2x). The water was then removed from the combined aqueous layers via rotary evaporation (heating to 55 °C). Acetone (13 V) was then added to the dried aqueous layer and the suspension was refluxed for 30 min – 1 hour (up to 1 hour for larger scale reaction). The suspension was allowed to cool to room temperature then 5 °C. The material was then filtered to yield white solid **10**.

3.2 Additional Filtration Information

Table S1. LCMS area percentages at 280 nm of the solid material that was filtered out of the reaction upon cooling was then dissolved in water to confirm its makeup: 90% compound **8** (ret. time 0.324 min) and 10% compound **10** (ret. time 1.082 min).

Signal 3: DAD1 G, Sig=280,4 Ref=360,100

Peak #	RetTime [min]	Type	Width [min]	Area [mAU*s]	Height [mAU]	Area %
1	0.324	BB	0.0503	1172.10840	377.01727	89.8024
2	1.082	BB	0.0925	133.10013	22.67731	10.1976
Totals :				1305.20853	399.69459	

3.3 Additional Purification Information

Table S2. Cytidine acylation reaction and purification information. Isolated yields as well as purification type used is described for the different scale reactions performed.

**8** Scale (g)	Solvent vol.	enzyme wt.%	Oxime ester **9** equiv	Reaction Time (hours)	Product **10** LC area%[a]	Diacylated **14** LC area%[a]	Product **10** IY	Product recovery (based on LC area%[a])
20	40 V	150	4.0	38	65%	10%	60% (>99% purity)	92%
30	50 V	150	4.0	40	73%	11%	65% (>99% purity)	89%
100	52.5 V	150	3.2	43	88%	4%	68% (98% purity)	77%
100	60 V	150	3.2	43	86%	9%	70% (>99% purity[b])	81%
19	50 V	150	4.0	42	73%	15%	66% (>99% purity)	90%
200	52.5 V	150	4.0	43	86%	9%	70% (96% purity)	81%

[a]LCMS area % determined at 280 nm
[b] Purity Determined by HPLC at 280 nm

(yellow) = purification A
(green) = purification B
(pink) = purification C

Section 4: Transamination of 5’-*O*-isobutyrylcytidine 10

4.1 Optimization of Transamination Reaction

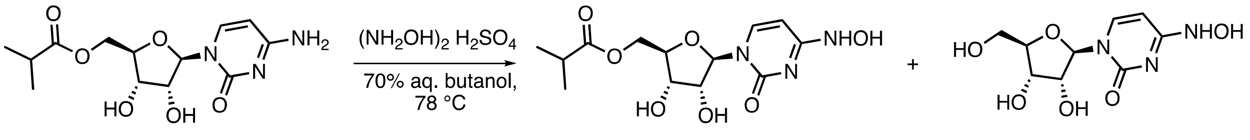

10 **7** **13**

Scale of **10**	$(NH_2OH)_2\ H_2SO_4$ equiv	time (h)	LCMS conversion[a] (**10**/**7**/**13**)	**7** crude purity[b]
1 g	4.5	22 h	9.6 / 88.8 / 1.6	73%
1 g	3	22 h	8.1 / 89.6 / 1.6	85%
1 g	2	22 h	8.7 / 89.1 / 1.5	84%
5 g	3	22 h	5.8 / 91.8 / 1.7	97%

[a]Conversion ratios determined at 280nm; [b]Purity determined by qNMR

Figure S14. Screening stoichiometry of $(NH_2OH)_2\ H_2SO_4$ to determine conversion as well as increase crude product (**7**) purity.

Table S3. Purity of crude reaction as well as after recrystallization in water

Scale of **10**	$(NH_2OH)_2\ H_2SO_4$ equiv	time (h)	LCMS conversion[a] (**10**/**7**/**13**)	**7** crude purity[b]	**7** isolated yield (purity[b])
10 g	3	24 h	3.6 / 93.6 / 2.1	92%	48% g (>99%)
20 g	3	28 h	1.5 / 95.6 / 1.5	93%	49% (>99%)
80 g	3.2	40 h	3.5 / 90.9 / 1.5	80%	58% (97%)

[a]Conversion ratios determined at 280nm; [b]Purity determined by qNMR

Section 5: NMR Spectra

5.1 Compound 11

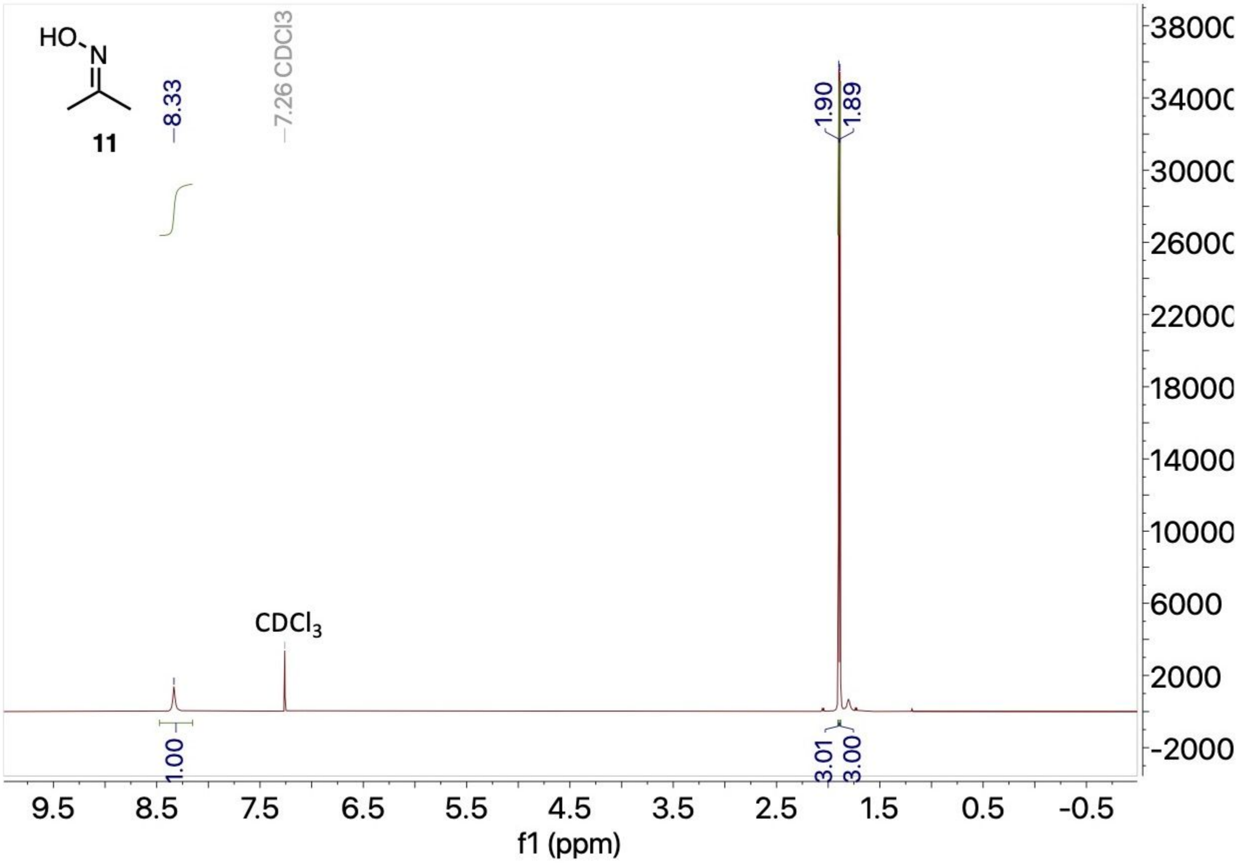

Figure S15. ^{1}H NMR Spectrum of compound 11 (400 MHz, $CDCl_3$).

5.2 Compound 9

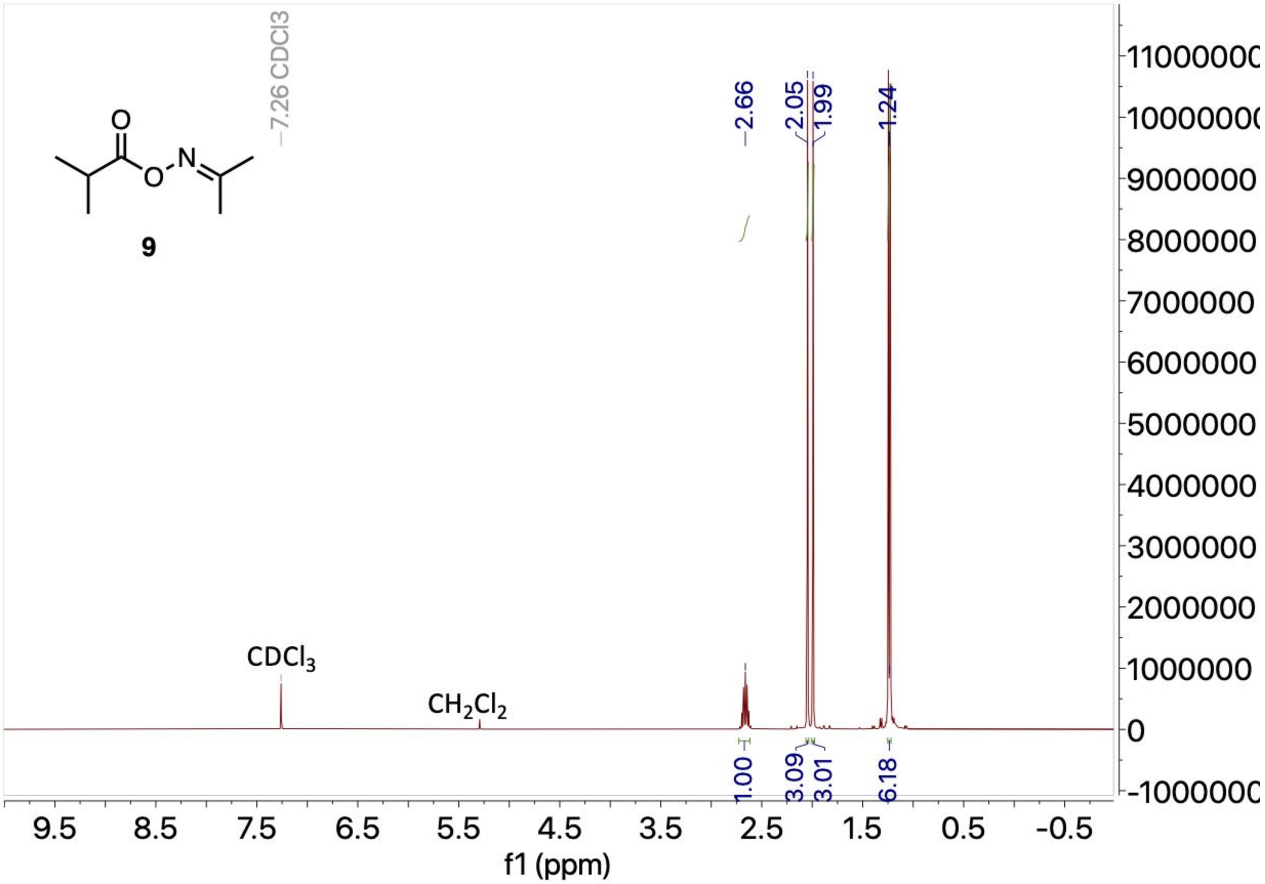

Figure S16. ^{1}H NMR Spectrum of compound 9 (400 MHz, $CDCl_3$).

5.3 Quantitative ^{1}H NMR of Compound 10

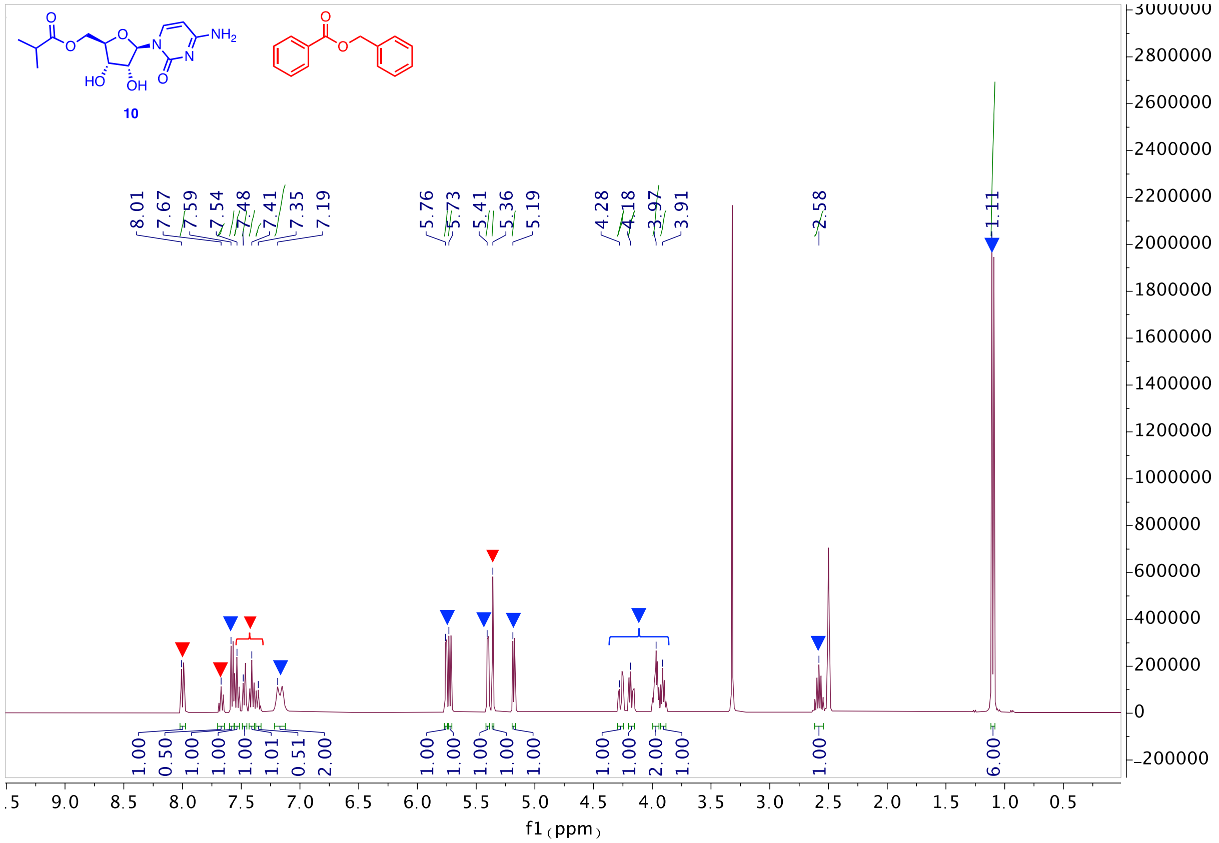

Figure S17. ^{1}H qNMR Spectrum of compound 10 with benzyl benzoate as internal standard (1.0:0.5, **10**:benzyl benzoate) (400 MHz, DMSO-d_6).

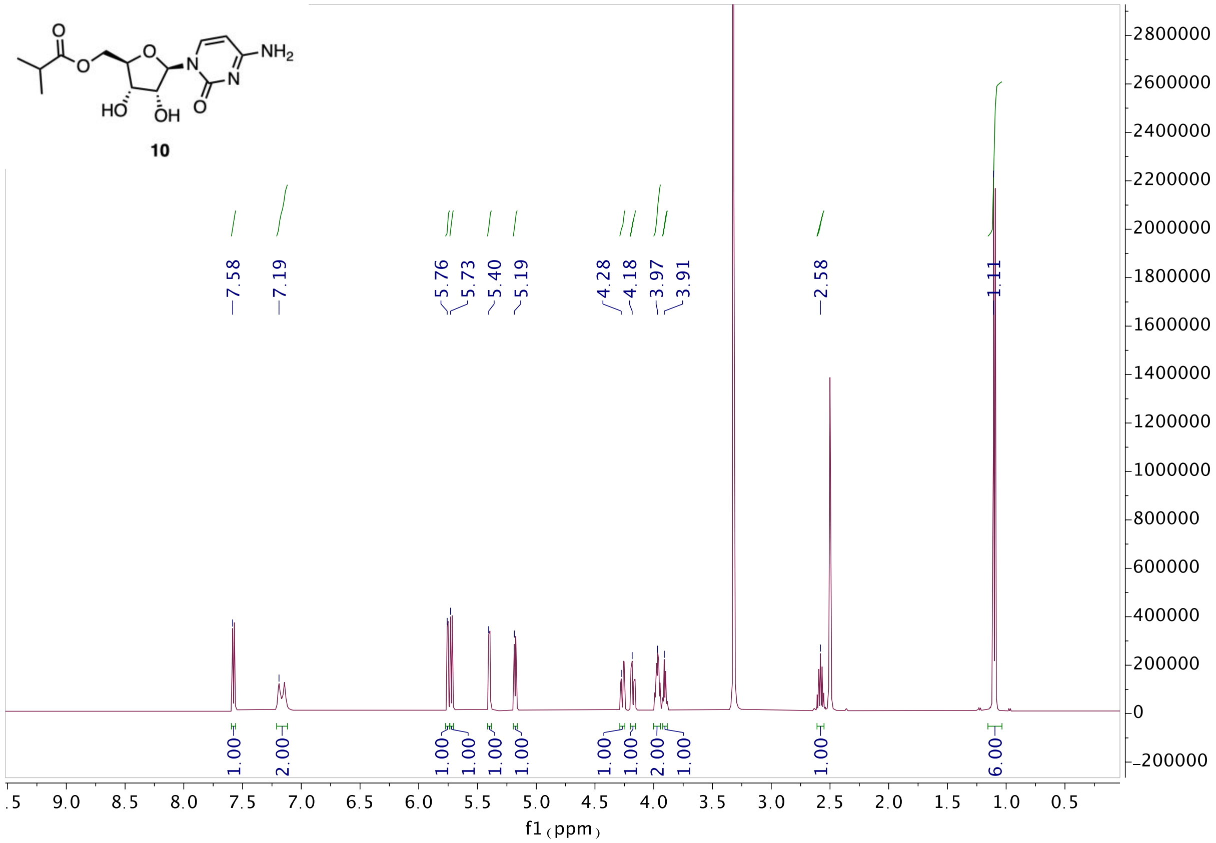

Figure S18. ^{1}H qNMR Spectrum of compound **10** (500 MHz, DMSO-d_6).

5.4 Quantitative ^{1}H NMR of Compound 7

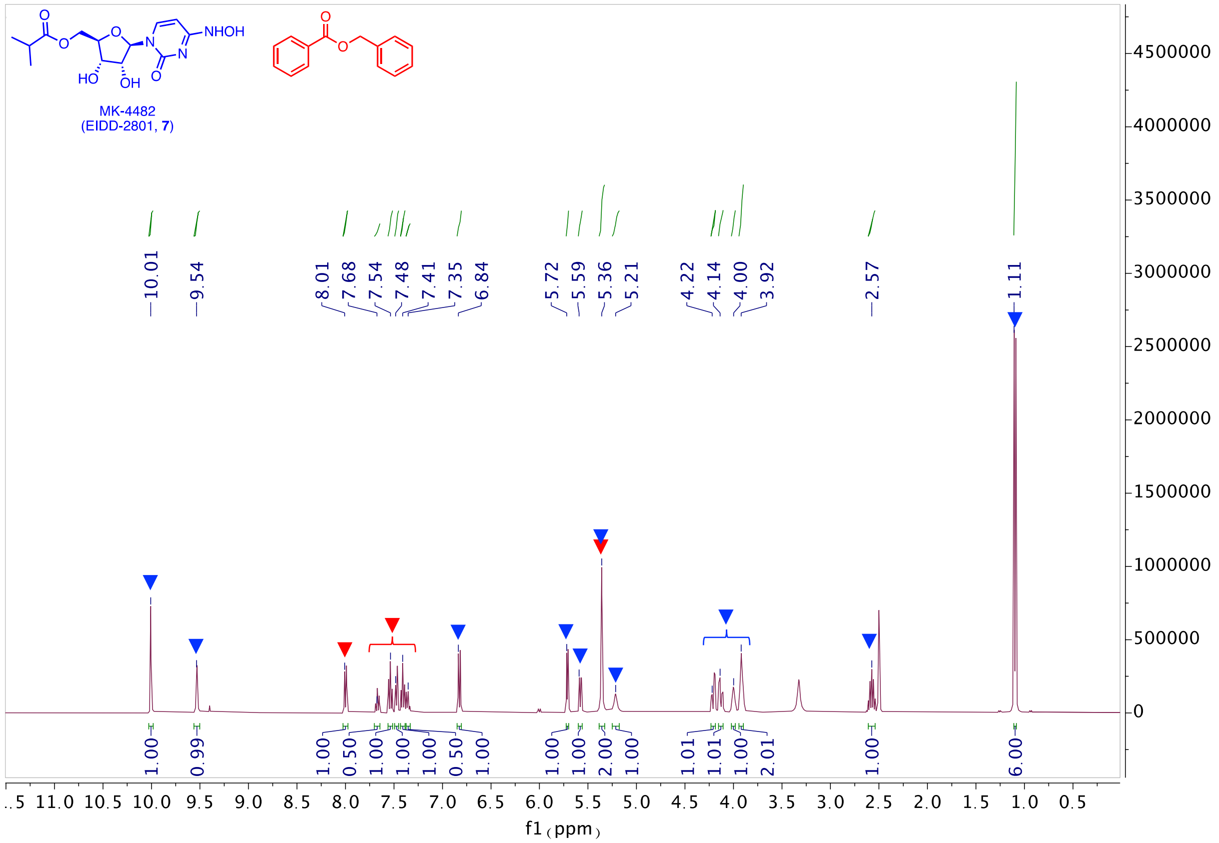

Figure S19. ^{1}H qNMR Spectrum of compound **7** with benzyl benzoate as the internal standard (1.0:0.5, **7**:benzyl benzoate) (400 MHz, DMSO-d_6).

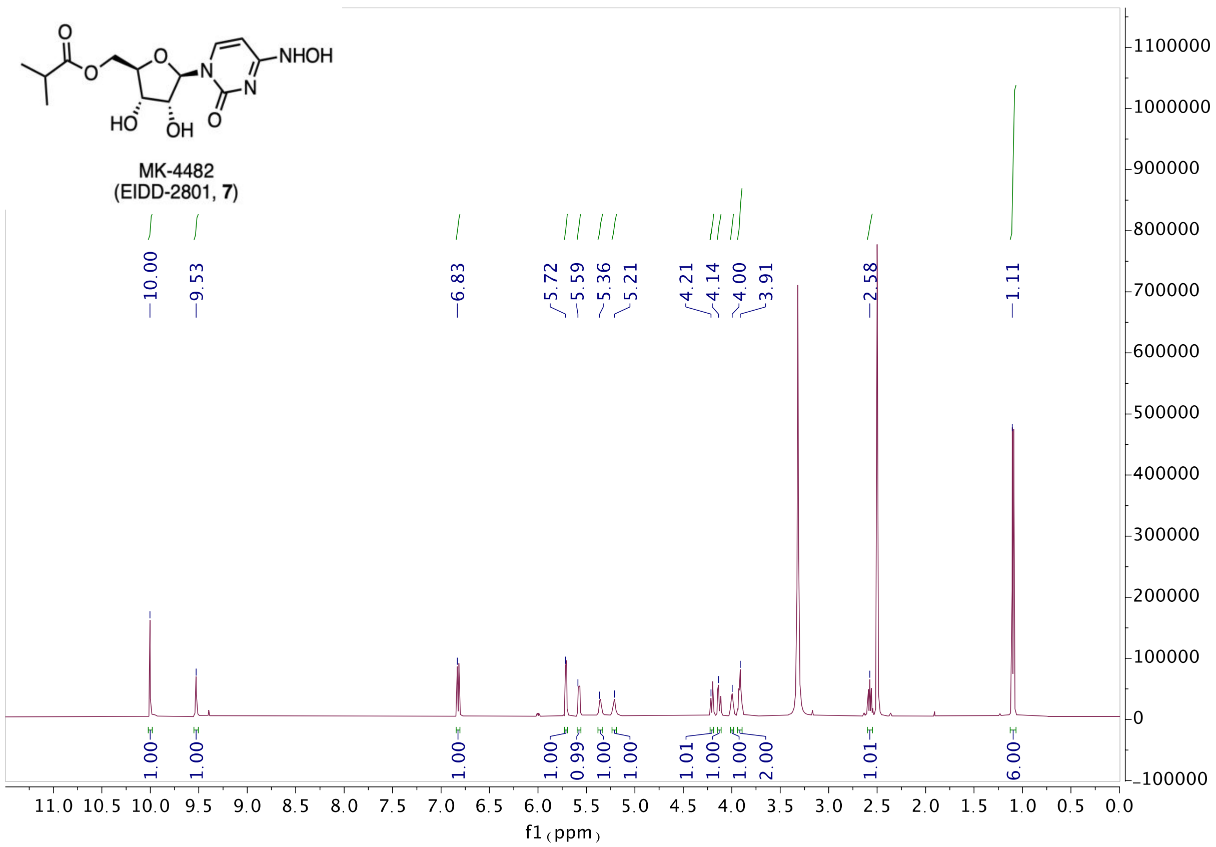

Figure S20. ^{1}H qNMR Spectrum of compound **7** (500 MHz, DMSO-d_6).
